# Toward non-invasive early pest surveillance: cross-modal adaptation using PLMS acoustic-visual representation and pre-trained transfer learning

**DOI:** 10.3389/fpls.2025.1610163

**Published:** 2025-10-02

**Authors:** Yaqin Wu, Lijun Cheng, Wangli Hao, Jianjun Niu, Yuze Li, Yan Chang, Jia Lv, Xuru Li

**Affiliations:** School of Software, Shanxi Agricultural University, Jinzhong, Shanxi, China

**Keywords:** pest surveillance, insectsound1000, acoustic-visual, PLMS, transfer learning

## Abstract

Pest infestations pose significant threats to agricultural productivity and ecological balance, making early prevention crucial for effective management. Toward non-invasive early-stage pest surveillance, this study introduces a novel cross-modal adaptation paradigm, leveraging the comprehensive bioacoustic repository, InsectSound1000 database. Firstly, the methodology initiates with adaptive audio preprocessing, where raw signals are filtered using the low-pass filter to remove high-frequency interference, followed by the downsampling operation to prevent aliasing and reduce computational complexity. Secondly, Patch-level log-scale mel spectrum (PLMS) spectrograms are proposed to convert acoustic signals into visual representations, refining time-frequency patterns through patch-level hierarchical decomposition to capture low-frequency and localized spectral features. The logarithmic transformation further enhances subtle low-frequency insect sound characteristics, optimizing feature analysis and boosting model sensitivity and generalization. Next, the PLMS acoustic-visual spectrograms undergo data augmentation prior to being processed by the pre-trained You Only Look Once version 11(YOLOv11) model for deep transfer learning, facilitating the efficient extraction of high-level semantic features. Finally, we compare the proposed algorithm with traditional acoustic features and networks, investigating how to balance preserving the frequency content of the signal and meeting computational requirements through optimized downsampling. Experimental results demonstrate that the proposed method achieves an Accuracy@1 of 96.49%, a Macro-F1 score of 96.49%, and a Macro-AUC of 99.93% at the 2500Hz sampling rate, showcasing its superior performance. These findings indicate that cross-modal adaptation with PLMS spectrograms and YOLOv11-based transfer learning can significantly enhance pest sound detection, providing a robust framework for non-invasive, early-stage agricultural pest surveillance.

## 1 Introduction

Agriculture plays a critical role in the global economy, food security, and rural development. As the backbone of many nations, particularly in developing regions, agriculture supports the livelihoods of billions of people and is responsible for producing food, fiber, and raw materials that sustain both local and global markets (FAO, 2024). Meanwhile, the increasing significance and attention towards sustainable agriculture as a solution to global challenges and a driver of rural development (Rusdiyana et al., 2024). However, the sector faces significant challenges, especially due to climate change, population growth, pest invasion and the increasing demand for sustainability. Climate change, in particular, has a significant negative impact on agricultural productivity (Bai et al., 2024). Concurrently, the rapidly growing global population intensifies concerns over food security. By 2050, the world will need to feed approximately 10 billion people, without depleting the planet’s resources or damaging the environment (Ghosh et al., 2024). Consequently, ensuring food security while preserving environmental sustainability has emerged as one of the most pressing challenges of the 21st century (Varzakas and Smaoui, 2024). Moreover, the escalating frequency and severity of pest invasions, frequently exacerbated by climate variability and global trade, further jeopardize crop yields and compromise the stability of agricultural systems worldwide. Among these challenges, pest infestation is a critical concern, as it directly impacts crop yields, food security, and the livelihoods of millions of farmers. To address this, Integrated Pest Management has emerged as a sustainable solution, effectively minimizing reliance on pesticides while simultaneously improving crop productivity and promoting ecosystem health (Zhou et al., 2024).

Pest infestation is critical to sustaining agricultural productivity. Certain insects, rodents, and other pests inflict significant damage to crops, resulting in major economic losses and jeopardizing food security. In addition to direct agricultural impacts, pest infestations can have profound environmental and social consequences. Environmentally, pests may lower biodiversity, endanger local species through predation and competition, destroy habitats, and interfere with pollination and other ecological processes. Socially, pests threaten food security, which can result in starvation and social unrest, as well as harming urban surroundings and cultural heritage, like when invasive pests cause urban trees to disappear. Furthermore, the health and well-being of communities can also be impacted by pests; urban pests like bedbugs and rats frequently indicate underlying psychological, social, or economic problems in local communities. Effective pest management strategies aim to promote sustainable agricultural practices, significantly reduce reliance on synthetic pesticides, and address a range of socio-economic, environmental, and human health challenges (Deguine et al., 2021). Although traditional pest control methods, such as chemical pesticides, have been widely adopted, their long-term adverse effects, including pesticide resistance, environmental degradation, and harm to non-target species, are becoming increasingly apparent (Gandara et al., 2024; Liu et al., 2024; Liu et al., 2024). Consequently, there is an urgent need for more sustainable and efficient pest control alternatives.

Among the various methods developed, image-based pest detection and control systems have garnered significant attention, driven by recent advancements in computer vision and machine learning techniques. For example, Li et al. (2021) explores the technical methods and frameworks of deep learning for smart pest monitoring, focusing on insect pest classification and detection based on field images. The study provides a comprehensive analysis of methodologies across key stages, including image acquisition, data preprocessing, and modeling techniques. By analyzing the captured images, the system could accurately identify the presence and quantity of pests, enabling farmers to take appropriate control measures promptly. However, image-based methods also face inherent challenges, including the lack of large, well-annotated image datasets, the impact of environmental factors on insect features, and difficulties in detecting hidden or camouflaged pests arising from their position and similarity to other species (Ngugi et al., 2021). Additionally, these systems may require substantial computational resources and be sensitive to environmental factors such as lighting and weather conditions, all of which complicate AI-based approaches (Kiobia et al., 2023).

Given these limitations, researchers have been exploring alternative and complementary techniques, one of which is pest detection through acoustic recognition technology. Unlike image-based methods that rely on visual cues, acoustic-based detection utilizes the unique acoustic features emitted by pests during behaviors such as feeding, mating, or movement, offering a promising avenue for accurate identification and monitoring. For example, the chirping of *crickets* or the buzzing of certain *beetles* can be distinct identifiers. Moreover, acoustic technology offers valuable insights into stored insect behavior, physiology, abundance, and distribution, providing information that is otherwise challenging to obtain through traditional methods (Mankin et al., 2021). To build upon this, acoustic technology can operate effectively in complete darkness or in environments with dense foliage, where visual access is restricted. Unlike visual methods, it is unaffected by variations in lighting that may degrade image quality. Moreover, the hardware required for sound acquisition, such as basic microphones, is often more cost-effective than high-resolution cameras. However, acoustic detection can be susceptible to environmental noise and adverse weather conditions such as wind, rain, or foliage movement, which may mask or distort target acoustic signals. Following this line of research, a low-cost real-time platform for the acoustic detection of *cicadas* in plantations was introduced (Escola et al., 2020). Similarly, the system proposed (Ali et al., 2024), driven by Internet of Things-based (IoT-based) computerized components, utilized machine learning on insect acoustic recordings, further enhancing the accuracy and reliability of pest detection. Thus, acoustic-based pest detection provides an effective, cost-efficient, and adaptable solution for pest monitoring across diverse environments.

In this study, a novel approach for early prevention of pest infestation is proposed, utilizing a cross-modal adaptation framework based on the InsectSound1000 database. The main contributions of the proposed approach are summarized as follows:

- We introduced an advanced methodology for transforming one-dimensional time-series signals into two-dimensional PLMS spectrograms, facilitating a more structured and informative acoustic-visual representation of insect acoustic characteristics.
- We leveraged transfer learning by fine-tuning the pre-trained YOLOv11 model, capitalizing on its robust feature extraction capabilities acquired from large-scale datasets after data Augmentation.
- We optimized computational efficiency by fine-tuning only a subset of model parameters, minimizing Floating Point Operations Per Second (FLOPS) and parameter count to achieve the lowest resource footprint among compared models, enabling real-time pest surveillance.
- We explored the impact of different patch sizes and sample rates on classification performance, investigating the optimal parameter tuning to balance preserving the frequency content of the signal while meeting computational requirements.
- Extensive experiments were conducted using the InsectSound1000 dataset. Results demonstrate that our method achieves superior classification performance, significantly outperforming existing related works.

## 2 Related works

A substantial cohort of researchers conducts investigations on acoustic-based insect detection. As shown in **Table 1**, this section provides a comprehensive synthesis of the relevant literature in terms of methodology, dataset, strengths and limitation to provide context for the present study.

**Table 1:** A concise overview of the literature reviews.

Methodology	Dataset	Strengths	Limitations	Ref.
Transformer-based networks with data augmentation	518 audio samples of 15 *bee* species	F1: 64.5%, Accuracy: 82.2%	Small dataset; Class imbalance; Reliance on pre-training	(Ferreira et al., 2025)
DFSM with Efficientnet and dual towers	InsectSet32	Accuracy: 80.26%, outperforms SOTA by 3%	Class imbalance; Insufficient data; Unaddressed real-world issues (insect sound variability and scalability for large-scale pest monitoring)	(He et al., 2024)
ML algorithms with MFCC features and data augmentation	Sound recordings of *cicada, beetle, termite*, and *cricket*	Improved generalization, reduced overfitting, diverse data augmentation	Potential over-reliance on augmentation; Limited to four insect types	(Wang and Vhaduri, 2024)
MEMS microphone, multi-layer CNN	Sounds of *lesser grain borer, rice weevil, and red flour beetle* in stored paddy grains	Accuracy: 84.51%, non-chemical pest detection, high information density handling	Limited to adult insect stages, potential noise interference in storage environments; Lack of diversity	(Balingbing et al., 2024)
Hyper-parameter tuning, MFCC	BUZZ1, BUZZ2, and add_BUZZ2	Accuracy: 96.9%	Limited to *bee* buzzing recognition	(Phan et al., 2023)
Sound-to-image conversion, feature fusion, DL classification	Recordings from date palm trees in Al-Ahssa, Saudi Arabia	Outperformed existing techniques for public datasets	Limited to *red palm weevil* infestation classification	(Boulila et al., 2023)
UAV visual-acoustic system, DL source separation, spectral denoising, CNN transfer learning	100+in-field 48 Megapixels(MP) photos, 16 species audio recordings	Precision(visual: 0.92, acoustic: 0.87), Recall (visual: 0.84, acoustic: 0.90), cost-effective (<$1000/unit), covers 12,500 m²/hr	Limited to *grasshoppers*; Requires UAV deployment; Noise interference challenges	(Zhang, 2023)
MFCC features, CNN	2800 acoustic samples	Precision (positive: 0.89, negative: 0.98), Recall (positive: 0.98, negative: 0.90), F1 (positive:0.93, negative: 0.94)	Limited to *Rice Weevils*; Requires high-performance microphone	(Montemayor et al., 2024)
IoT and DMF-ResNet	Bug Bytes sound library	Accuracy (99.75%), Precision (99.18%), Recall (99.08%), F1 score (99.11%)	High initial cost; Limited to specific pests	(Dhanaraj et al., 2024)
LEAF and mel-spectrogram	InsectSet32 (32 species), InsectSet47 (47species), InsectSet66 (66 species); Focused on *Orthoptera* and *Cicadidae*	InsectSet32: LEAF Accuracy (78%); InsectSet47: LEAF Accuracy (86%); InsectSet66: LEAF Accuracy (83%)	Small dataset; Limited audio quality	(Faiß and Stowell, 2023)
CNN-GRU model with Mel Spectrogram and Bayesian Optimization	BUZZ1, BUZZ2, and add_BUZZ2	Outperforms existing models by 1% in bee sound identification.	Limited to *bee* buzzing sounds; Small improvement margin (1%).	(Truong et al., 2023)
ResNet-9	Wingbeats; Fruitfiles; Abuzz	High accuracy, reduced trainable parameters (90% reduction)	Limited to *fruit flies* and *mosquitoes*	(Szekeres et al., 2023)
Improved MFCC scanning with ML models	Collected from MobCup, Quick Sounds, and Pixabay; includes 9 insect species	Achieved 85.4% accuracy with kNN	Limited to 9 insect species	(Basak et al., 2022)
Empirical Mode Decomposition (EMD) and Paraconsistent Feature Engineering (PFE) for feature extraction, SVM for classification	1366 audio files (683 *cicada*, 683 noise) from São Paulo and Minas Gerais, Brazil	Accuracy (98%)	Limited to *Quesada gigas* species	(de Souza et al., 2022)
Syllable segmentation, Spectrogram representation, CNN	43 sound recordings of three *cicada* species	Accuracy (66.67% to 100%), robust species recognition	Small dataset; limited to *cicada* species; Dependent on syllable segmentation	(Tey et al., 2022)
IoT-based with fine-tuned InceptionResNet-V2	TreeVibes database (1754 samples: 1023 clean, 731 infested)	Accuracy (97.18%), effective transfer learning, real-time detection	Limited to *Red Palm Weevils*; Small dataset	(Karar et al., 2021)
MFCC feature, CNN	Insect sound library from ARS Center	Accuracy (92.56%)	Limited dataset	(Zhang et al., 2021)
MFCC and LFCC	343 species of *katydids, crickets and cicadas*	Accuracy (98.07%)	Limited to 3 species	(Noda et al., 2019)
Enhanced spectrogram, CNN	47 types of insect sounds from USDA library	Accuracy (97.87%), reduced data size, and faster training	Small dataset	(Dong et al., 2018)
MFCC, Bagged Tree, KNN	11 insects from 6 species	species classification (over 97.1%); insect classification (over 92.3%)	Limited sample size; Short-term features only	(Phung et al., 2017)
MFCC and LFCC, SVM	InsectSingers	Accuracy (99.08%)	Limited to *cicada* species	(Noda et al., 2016)
MFCC, Probabilistic Neural Network	insect sound library from agricultural research service of United States department of agriculture	Accuracy (96%)	Limited to 6 species	(Le-Qing, 2011)

The methodologies employed in insect acoustic recognition and detection can be broadly categorized into traditional machine learning (ML) and deep learning (DL) techniques. Traditional ML methods, as exemplified by studies such as references (Le-Qing, 2011; Noda et al., 2016; Phung et al., 2017; Noda et al., 2019; Basak et al., 2022; de Souza et al., 2022; Wang and Vhaduri, 2024), rely on handcrafted features like mel frequency cepstral coefficients (MFCC) and linear frequency cepstral coefficients (LFCC), paired with classifiers such as support vector machines (SVM) and k-Nearest Neighbors (kNN). These approaches have achieved notable accuracy in constrained scenarios, for instance, Phung et al. (2017) reported 97.1% accuracy for 11 insect species, while Noda et al. (2016) achieved 99.08% accuracy for cicada detection. However, their reliance on manual feature engineering limits adaptability to complex or high-frequency acoustic patterns, as highlighted by the analysis (Le-Qing, 2011; Phung et al., 2017).

In contrast, DL techniques leverage automated feature learning and scalable architectures to overcome these limitations. Studies such as those in references (Karar et al., 2021; Zhang et al., 2021; Tey et al., 2022; Boulila et al., 2023; Faiß and Stowell, 2023; Szekeres et al., 2023; Truong et al., 2023; Zhang, 2023; Balingbing et al., 2024; Dhanaraj et al., 2024; Montemayor et al., 2024; Ferreira et al., 2025) employ convolutional neural networks (CNNs), gated recurrent units (GRUs), transformers, and hybrid models, often integrated with IoT or unmanned aerial vehicle (UAV) systems. For instance, the deep multibranch fusion residual network (DMF-ResNet) (Dhanaraj et al., 2024), trained on the comprehensive “Bug Bytes sound library”, demonstrated exceptional performance with 99.75% accuracy, 99.18% precision, and 99.08% recall, showcasing the potential of DL for high-precision applications. Similarly, Kamar et al. (Karar et al., 2021) utilized the IoT-based Inception-Residual Network V2 (InceptionResNet-V2) model on the TreeVibes dataset, achieving 97.18% accuracy for real-time red palm weevil detection while addressing class imbalance through transfer learning. UAV-integrated systems (Zhang, 2023) further expanded scalability by combining 48MP visual data with acoustic recordings from 16 species to achieve 92% precision and 84% recall for large-area *grasshopper* monitoring. Adaptive frontends, such as the learnable frontend (LEAF) model (Faiß and Stowell, 2023), dynamically adjusted filter parameters for high-frequency sounds, leading to an accuracy boost from 67% to 86% across multiple datasets, thereby outperforming static Mel-spectrogram method. Similarly, the CNN-Gated Recurrent Unit (GRU) model (Truong et al., 2023) enhanced bee buzzing recognition by 1% over existing methods, leveraging Bayesian optimization for hyperparameter tuning. Despite these advancements, DL models still face several challenges. One major issue is computational intensity, as evidenced by the IoT system (Dhanaraj et al., 2024), which requires significant resources. Another challenge is dataset dependency, highlighted by the transformer model (Ferreira et al., 2025), which necessitated data augmentation for 15 *bee* species. However, reliance on augmentation introduces risks, particularly the potential for overfitting to synthetic variations, as noted in Wang et al (Wang and Vhaduri, 2024). Additionally, environmental noise sensitivity remains a concern, as seen in the UAV recordings (Zhang, 2023), which suffered from interference.

The strengths and limitations of these methodologies are intricately shaped by the inherent characteristics of the datasets employed. Some studies focus on small, specialized datasets, such as the 43 acoustic recordings of three *cicada* species (Tey et al., 2022), datasets focused on *fruit flies* and *mosquitoes* (Szekeres et al., 2023), *bee* buzzing sounds (Phan et al., 2023), and 343 samples spanning three insects (Noda et al., 2019). While these datasets are useful for specific applications, they often lack the diversity required for robust generalization. Larger datasets, such as the TreeVibes database (Karar et al., 2021) and the InsectSet66 dataset (Faiß and Stowell, 2023), offer a more comprehensive coverage of insect sounds. However, even these datasets face challenges, such as class imbalance and limited audio quality (Karar et al., 2021; Faiß and Stowell, 2023). On the other hand, several studies highlighted limitations in scalability and dependency on specialized equipment. For instance, the dual-frequency and spectral fusion module (DFSM) architecture with EfficientNet (He et al., 2024) achieved 80.26% accuracy on InsectSet32, outperforming state-of-the-art (SOTA) methods by 3%, but scalability for large-scale monitoring remained an unresolved issue. Similarly, Zhang et al. (Montemayor et al., 2024) achieved high precision and recall using MFCC features and CNNs on 2,800 *rice weevil* samples but required high-performance microphones, limiting practical deployment. Innovations like micro-electromechanical system (MEMS) microphones (Balingbing et al., 2024) enabled non-chemical pest detection with 84.51% accuracy but faced challenges with noisy storage environments and limited taxonomic coverage.

## 3 Data description

The comparison of various insect sound datasets is presented in **Table 2**, highlighting the species covered, sample sizes, descriptions, and download links. These datasets differ in terms of the number of species, total samples, and species diversity. In comparison, datasets such as BUZZ1 (Kulyukin et al., 2018), BUZZ2 (Kulyukin et al., 2018), SINA (Walker and Moore, 2019), and Insectsingers (Marshall and Hill), focus on a limited number of insect species, resulting in less diversity in terms of species variety and sound level range. Additionally, datasets like ESC-50 (Piczak, 2015), and InsectSet32 (Faiß, 2022), focus on fewer species with smaller sample sizes. The Bug Bytes sound library (Mankin, 2019), features a broader range of insect species (72), with 7,200 samples. However, the presence of non-agricultural pest species may introduce interference, limiting the dataset’s effectiveness for agricultural pest monitoring.

**Table 2:** Comparison of various insect sound datasets.

References	Insect species	Number of samples	Brief description
BUZZ1 (Kulyukin et al., 2018) Available online: https://usu.app.box.com/v/BeePiAudioData (accessed on 11 May 2021).	*Bee\Cricket\Noise*	3300\3500\3460	Very few insect species
BUZZ2 (Kulyukin et al., 2018) Available online: https://usu.app.box.com/v/BeePiAudioData (accessed on 11 May 2021).	*Bee\Cricket\Noise*	4300\4500\4114	Very few insect species
SINA (Walker and Moore, 2019) Available online: https://orthsoc.org/sina/crickets.htm. (accessed on 7 September 2025)	255 species of *katydids, crickets* and *cicadas*	/	Only three insect species
Insectsingers (Marshall and Hill) Available online: https://www.insectsingers.com/. (accessed on 7 September 2025)	343 species of *katydids, crickets* and *cicadas*	/	Only three insect species
ESC-50 (Piczak, 2015) Available online: https://github.com/karolpiczak/ESC-50?tab=readme-ov-file. (accessed on 7 September 2025)	*Frog*\Insects(flying)\*Criets*\*Chirping birds*	40 examples per class, each 5 seconds long	No subdivision of insect species
InsectSet32 (Faiß, 2022) Available online: https://doi.org/10.5281/zenodo.7072196. (accessed on 7 September 2025)	9 species of *Orthoptera* and 23 species of *Cicadidae*	335 files, 57 minutes in total	Only two insect species
Bug Bytes sound library (Mankin, 2019) Available online: https://data.nal.usda.gov/dataset/bug-bytes-sound-library-stored-product-insect-pest-sounds. (accessed on 7 September 2025)	72	7200	Includes many non-agricultural pest insects, with diverse categories that may cause interference
InsectSound1000 (Branding et al., 2024) Available online: https://www.openagrar.de/receive/openagrar_mods_00091171. (accessed on 7 September 2025)	*Aphidoletes aphidimyza, Myzus persicae*, et al., a total of 12 species.	Over169,000 labelled samples	Diverse insect species with a wide range of sound levels.

Notably, InsectSound1000 (Branding et al., 2024) stands out as the most comprehensive and diverse dataset for training robust insect acoustic recognition models, especially given its extensive sample size and wide range of insect species and sound levels. Therefore, InsectSound1000 is selected for this study, containing over 169,000 labelled sound samples from 12 insect species, recorded in an anechoic box with a four-channel low-noise microphone array. The acoustic intensity spans a range from the very loud *Bombus terrestris* to the nearly inaudible *Aphidoletes aphidimyza* for the human ears. Each sample is a four-channel WAV file with a duration of 2500 ms, sampled at 16 kHz with 32-bit resolution. With over 1000 hours of high-quality recordings, InsectSound1000 is suitable for training DL models for insect acoustic recognition. Primarily used for model pre-training, this dataset also supports developing insect acoustic recognition systems across different hardware platforms for various species. As outlined in **Table 3**, the details of the pests used in the analytical procedures are provided. To ensure data balance and consistency, the final dataset is determined based on the class with the smallest sample size.

**Table 3:** Details of Pest used in analytical procedures.

Insect order	Insect family	Insect species	Number	Duration /ms	Brief description
*Diptera*	*Cecidomyiidae*	*Aphidoletes aphidimyza*	14065	2500	A natural predator of *aphids*, whose sound can be used for pest control monitoring.
*Hymenoptera*	*Apidae*	*Bombus terrestris*	18291	2500	A *bumblebee* species, whose buzzing sounds can be used to monitor pollinator activity and assess crop health.
*Diptera*	*Sciaridae*	*Bradysia difformis*	11394	2500	A small *fungus gnat*, whose larvae feed on *fungi* and damage the root systems of host plants in humid environments.
*Coleoptera*	*Coccinellidae*	*Coccinella septempunctata*	14682	2500	A beneficial *ladybug* that feeds on *aphids* and helps control agricultural pests.
*Diptera*	*Syrphidae*	*Episyrphus balteatus*	16868	2500	A *hoverfly* species, whose larvae feed on *aphids*, helping control agricultural pests.
*Heteroptera*	*Pentatomidae*	*Halyomorpha halys*	19671	2500	A destructive *stink bug*, whose feeding on plant tissues causes damage to crops, leading to yield loss and quality degradation.
*Hemiptera*	*Aphididae*	*Myzus persicae*	3208	2500	A destructive *aphid* that feeds on various crops, causing poor growth, yellowing leaves, and spreading plant viruses.
*Heteroptera*	*Pentatomidae*	*Nezara viridula*	20323	2500	A widespread agricultural pest that feeds on crops, causing damage to plant tissues and reduced yield.
*Heteroptera*	*Pentatomidae*	*Palomena prasina*	27340	2500	A *shield bug* that feeds on plant tissues, causing minor damage to crops and deformed fruits.
*Heteroptera*	*Pentatomidae*	*Rhaphigaster nebulos*	13443	2500	A *shield bug* that can cause minor damage to crops by feeding on plant tissues in certain circumstances.
*Hemiptera*	*Aleyrodidae*	*Vaporariorum*	1062	2500	A common greenhouse pest that damages plants by feeding on sap and can spread viruses, negatively affecting crop growth and yield.
*Lepidoptera*	*Gelechiidae*	*Tuta absoluta*	633	2500	A destructive pest of *tomato* crops, whose larvae damage leaves and fruits, leading to significant yield and quality losses.

## 4 Proposed methodology

**Figure 1:**
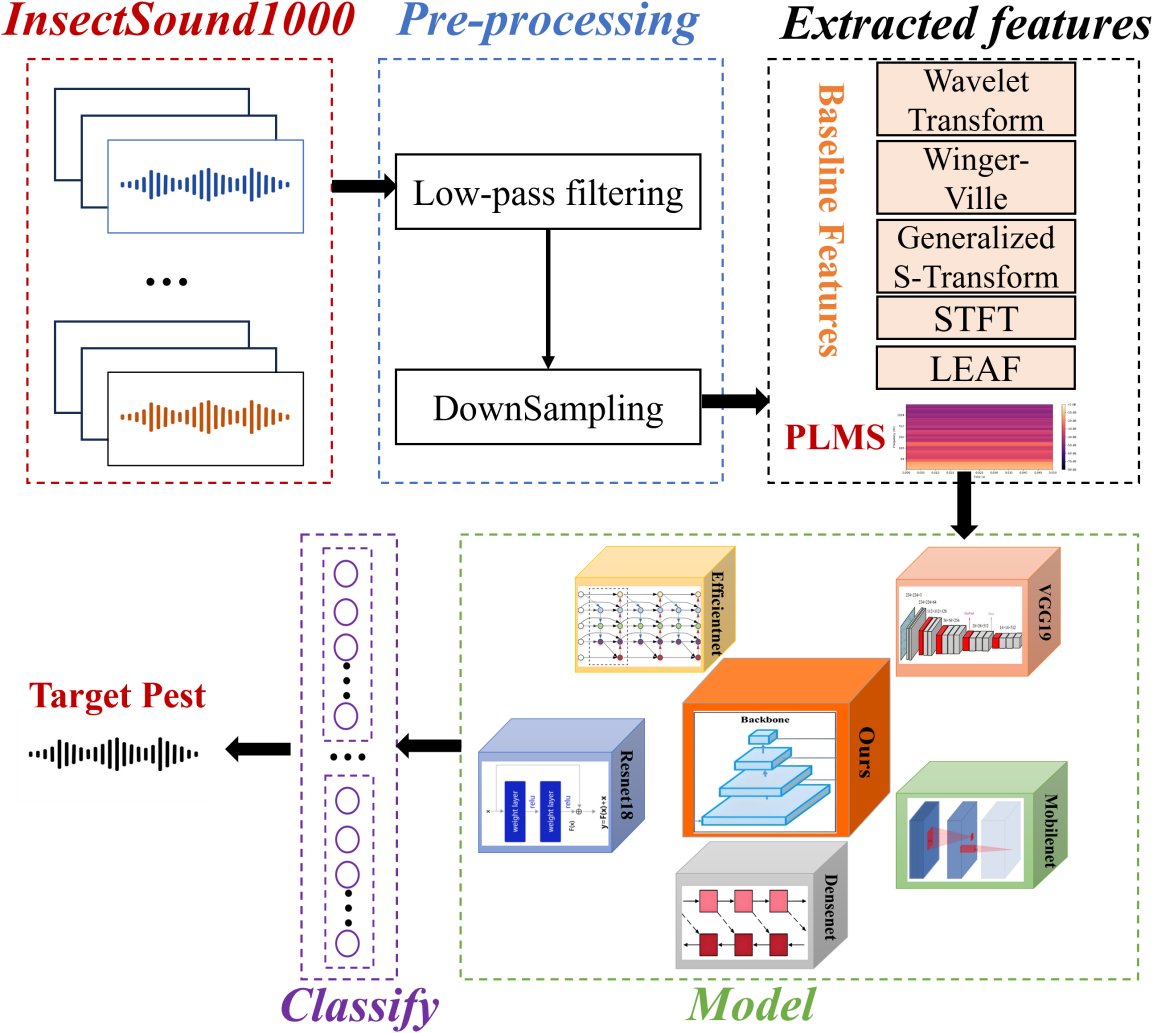
Architecture of the proposed approach for insect acoustic classification with comparative analysis of multiple features and benchmark models.

The objective of this study is to rapidly identify and detect pests at their early stages of appearance or as soon as they reach detectable levels, enabling the swift initiation of localized control measures to prevent further spread and minimize damage. For instance, upon detecting a small number of pests in a specific area of an orchard, the affected zone is immediately isolated. Subsequently, biological control methods or precision pesticide treatments are applied to eradicate the pests at this early stage, thereby preventing their spread to the entire orchard. **Figure 1** describes the architecture of the proposed approach. The process begins with pre-processing of the InsectSound1000 dataset, followed by PLMS feature extraction alongside conventional feature representations for comparative analysis. These feature representations are mapped into DL-based classification frameworks, where our proposed model is systematically benchmarked against ResNet18, EfficientNet, VGG19, DenseNet, and MobileNet and other algorithms to assess its efficacy and robustness. The resulting classification facilitates high-precision pest identification, enhancing automated surveillance and enabling proactive early-stage intervention strategies.

### 4.1 Preprocessing

#### 4.1.1 Low-pass filtering

**Figure 2:**
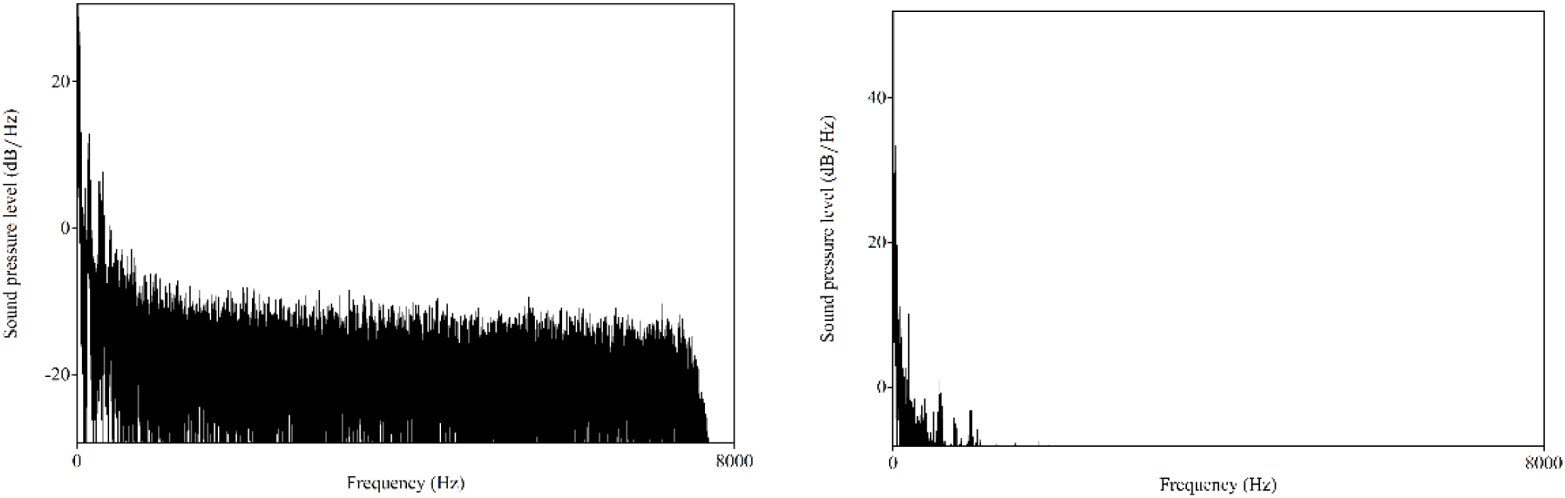
Spectra of the insect acoustic signals from *Aphidoletes aphidimyza* and *Bradysia difformis*.

The purpose of low-pass filtering is to eliminate components of the signal above specific frequency. **Figure 2** presents the representative spectrums of the insect acoustic signals from *Aphidoletes aphidimyza* and *Bradysia difformis*. Analysis shows that the acoustic signals of the pests studied in this paper are predominantly concentrated within the low-frequency range.

Therefore, finite impulse response (FIR) filter is employed for low-pass filtering to suppress high-frequency noise while effectively preserving the essential low-frequency information. FIR filters, a class of digital filters with finite-duration impulse responses, are distinguished by their ideal linear phase characteristics, where the output is computed as the convolution of the input signal with the filter coefficients, as depicted in Equation 1:


(1) $$y[n]=\sum_{k=0}^{N-1}h[k]x[n-k]$$

The discrete-time output of an FIR filter can be represented as the convolution of the input signal and the filter coefficients. Where *N* denotes the order of the filter, i.e. the number of filter coefficients. The design of an FIR filter typically involves three main steps: determining the filter type, specifying the cutoff frequency, and selecting an appropriate window function. In practical implementations, the coefficients of the FIR filter are commonly calculated using the window function method. This approach applies a window function to the impulse response of the ideal filter, effectively suppressing sidelobe leakage and ripple effects, thereby improving overall filter performance. In this study, low-pass filtering is selected. The time domain impulse response of an ideal low-pass filter is calculated as shown in Equation 2:


(2) $$h_{\mathrm{ideal}}\left(n\right)=\frac{\sin(2\pi f_{c}n)}{\pi n}$$

Where $f_{c}$ denotes the cutoff frequency. The ideal filter possesses an impulse response of infinite duration, rendering it infeasible for practical implementation. Therefore, it is necessary to truncate its duration through the window function method. The Hamming window can effectively reduce the sidelobe leakage and enhance the stopband attenuation performance. The application of the Hamming window yields a flatter frequency response within the passband, facilitates more rapid attenuation within the stopband, and effectively suppresses sidelobe levels. The Hamming window is calculated as shown in Equation 3:


(3) $$w[n]=0.54-0\text{.46cos(}\frac{2\pi n}{N-1}),0\leq n\leq N-1$$

The ideal impulse response is multiplied by the window function to obtain the final filter coefficients, as shown in Equation 4:


(4) $$h[n]=h_{\mathrm{ideal}}\left(n\right)\cdot w[n]$$

In addition, the choice of filter order is influenced by the transition band width, passband ripple, stopband attenuation, and other design parameters. A higher order results in a narrower transition band and more accurate frequency response, but it also increases computational complexity.

#### 4.1.2 Downsampling

In accordance with the Nyquist sampling theorem, the maximum frequency component of the signal must be less than half of the target sampling rate. If high-frequency components are not adequately attenuated prior to downsampling, aliasing artifacts may arise, wherein high-frequency energy is folded into the lower frequency spectrum, leading to significant distortion of the signal. Consequently, applying low-pass filtering before downsampling is essential to prevent aliasing effects.

Let $f_{\max}$ denotes the maximum frequency of the signal. The target sampling rate $f_{t}$ must satisfy the following condition, as shown in Equation 5:


(5) $$f_{t}\geq 2f_{\max}$$

Downsampling, an essential method in signal processing, is employed to reduce data dimensionality and mitigate computational load. Assume that the downsampling function is defined as *Resample*, which is defined as Equation 6:


(6) y'(n)=Resample(x(n))=x(n·fsft)=x(n/R)

Where $f_{s}$ denotes the sampling rate of the original signal x(n) . $f_{t}$ denotes the sampling rate of the target signal y'(n) . *R* denotes the sampling rate ratio. Since n/R is usually not an integer, interpolation must be performed. This paper utilizes the *sinc* interpolation method, a high-fidelity anti-aliasing resampling technique that integrates a window function to effectively suppress sidelobe leakage. The formula is defined as shown in Equation 7:


(7) y'(n)=∑kx(k)·sinc(nR−k)·w(k),  sinc(x)=sin(πx)πx

Where w(k) denotes the window function. The primary objective of downsampling is to reduce the number of sampling points, thereby lowering the computational burden associated with subsequent feature extraction and classifier model training. However, higher downsampling ratios may compromise temporal resolution, particularly affecting the accurate representation of high-frequency components. Therefore, the choice of an appropriate downsampling rate must achieve a trade-off between preserving the critical spectral characteristics of the signal and satisfying the computational efficiency requirements of downstream processing tasks.

### 4.2 Feature engineering

#### 4.2.1 PLMS

An innovative feature representation method, termed the PLMS, is proposed in this study to enhance the accuracy of insect sound signal recognition. The complete process of PLMS extraction is outlined in **Algorithm 1**. First, the original insect acoustic signals are preprocessed using low-pass filtering and downsampling to reduce computational complexity while preserving relevant spectral content. The processed signals are then segmented into overlapping patch-level windows with predefined window length and shift, enabling the extraction of local temporal structural features. Since these signals exhibit distinct spectral characteristics across different temporal regions, dividing the spectrogram into smaller, localized patches allows for the capture of subtle, context-specific variations in both time and frequency domains. This localized analysis enhances the model’s ability to detect fine-grained temporal dynamics, which is crucial for improving the accuracy and robustness of classification and recognition tasks. Furthermore, by enabling the model to learn discriminative features from multiple local regions, the patch-level approach contributes to better generalization performance when applied to unseen data. The specific formula for the patch-level segmentation operation is as shown in Equation 8:

Algorithm 1 **PLMS extraction.**

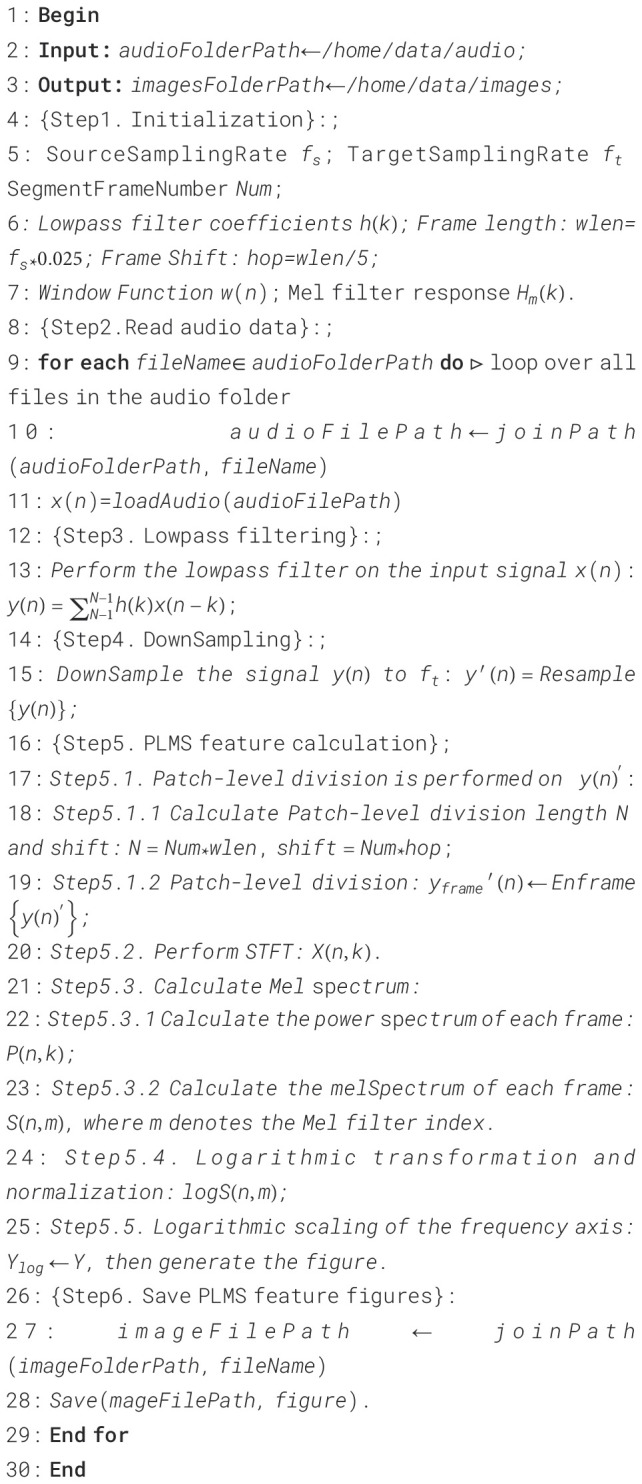

(8) y'frame(n)=Enframe(y'(n))=y'[n∗shift:n∗shift+N]

Where *N* and *shift* represent the patch-level window length and shift, respectively. Subsequently, the short-time fourier transform (STFT) is performed on each patch-level segmented signal to extract localized time-frequency features. This process facilitates the characterization of spectral variations within each temporal segment, thereby enhancing the representation of non-stationary signal components, as shown in Equation 9:


(9) $$X(n,k)=\sum_{m=0}^{N-1}\text{y}{'}_{\text{frame}}[n*shift+m]\cdot w(m)\cdot e^{-j2\pi km/N}$$

where *n* denotes the frame index. 𝑘 denotes the frequency index. X(n,k) denotes the STFT complex spectrum of the 𝑛-th frame.

Next, in order to compute the Mel spectrum for each frame, a bank of Mel-scale filters must first be constructed to project the power spectrum onto the perceptually motivated Mel frequency. The Mel filter bank comprises a series of triangular band-pass filters that are uniformly spaced on the Mel frequency scale but nonlinearly distributed along the linear frequency axis. Given *M* Mel filters, the frequency response *m*-th filter is defined as shown in Equation 10:


(10) $$H_{\text{m}}(k)=\left\{ \begin{array}{l} 0\;f_{k}<f_{m-1}\\ \frac{f_{k}-f_{m-1}}{f_{m}-f_{m-1}}\;f_{m-1}\leq f_{k}<f_{m}\;\\ \frac{f_{m+1}-f_{k}}{f_{m+1}-f_{m}}\;f_{m}\leq f_{k}<f_{m+1}\;\\ 0\;f_{k}\geq f_{m+1} \end{array}\right.\;$$

Where $f_{k}$ represents the k-th frequency point. $f_{m}$ represents the center frequency of the *m*-th Mel filter. $f_{m-1}$ and $f_{m+1}$ are the boundary frequencies of adjacent filters. Center frequency is usually mapped from the Mel scale to the frequency axis using the following formula as shown in Equation 11:


(11) $$f_{m}=f_{m\text{el}}^{-1}(\frac{m}{M+1}\cdot (f_{m\text{el}}(f_{\max})-f_{m\text{el}}(f_{\min}))+f_{m\text{el}}(f_{\min}))$$

Where the Mel transform is defined as shown in Equation 12:


(12) $$f_{m\text{el}}=2595\cdot \log_{10}(1+\frac{f}{700})$$

Then Mel filter bank and power spectrum are weighted superimposed frame by frame to obtain Mel spectrum, as shown in Equation 13:


(13) $$S(n,m)=\sum_{k=0}^{K-1}P(n,k)\cdot H_{m}(k)$$

Where P(n,k) denotes the power spectrum of each frame, computed as follows shown in Equation 14:


(14) $$P(n,k)=|X(n,k){|}^{2}$$

The Mel frequency scale, a nonlinear transformation of frequency, provides enhanced resolution during the low-frequency range while compressing resolution during the high-frequency domain. This property aligns well with the spectral characteristics of insect acoustic signals, where the energy is predominantly concentrated in the low-frequency components. To compress the dynamic range of the Mel spectrogram, the logarithmic transformation and normalization are applied, mitigating the influence of high-amplitude frequency components and highlighting finer details during low-energy regions, as shown in Equation 15:


(15) $$\log S(n,m)=10\cdot \log_{10}(\frac{S(n,m)}{ref}),\text{ ref}={\text{max}}_{n,m}S(n,m)$$

Finally, to align the visual representation of the Mel spectrogram with the logarithmic nature of human auditory perception, a logarithmic transformation is applied to its frequency axis. This scaling enhances the interpretability of spectral content, particularly in lower frequency regions where human sensitivity is greater. By mapping the linear frequency axis to a logarithmic scale, the resulting spectrogram more accurately reflects perceptual frequency resolution. Furthermore, this approach not only clarifies the low-frequency regions but also diminishes the visual dominance of high-frequency details, leading to a more balanced signal representation. Assume $f_{\log}$ is the transformed logarithmic frequency, the transformation formula is shown as Equation 16:


(16) $$f_{\log}=\log_{10}(f)$$

**Figure 3:**
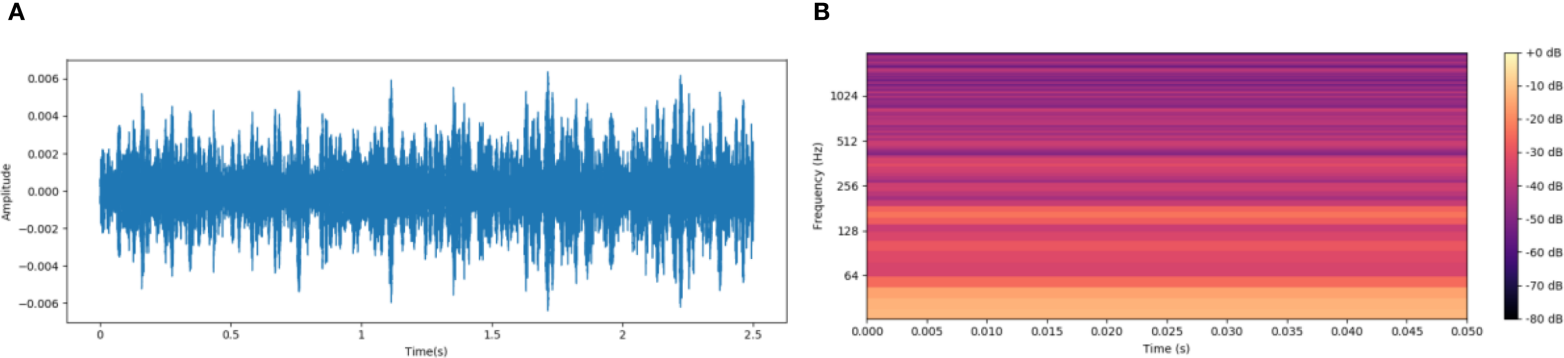
Comparison of original acoustic waveform and PLMS spectrogram. **(A)** Original audio waveform. **(B)** PLMS spectrogram.

From **Figure 3**, the original audio waveform (**Figure 3A**) and the PLMS spectrogram (**Figure 3B**) are presented for comparison. While the original waveform offers a broad, global representation of signal energy variations over time, it falls short in capturing the finer, localized details and intricate frequency characteristics inherent within the signal. In contrast, the PLMS provides a more refined and precise depiction by explicitly encoding time-frequency patterns through a hierarchical decomposition, thereby revealing essential spectral and temporal features that the waveform alone cannot fully convey.

**Figure 4:**
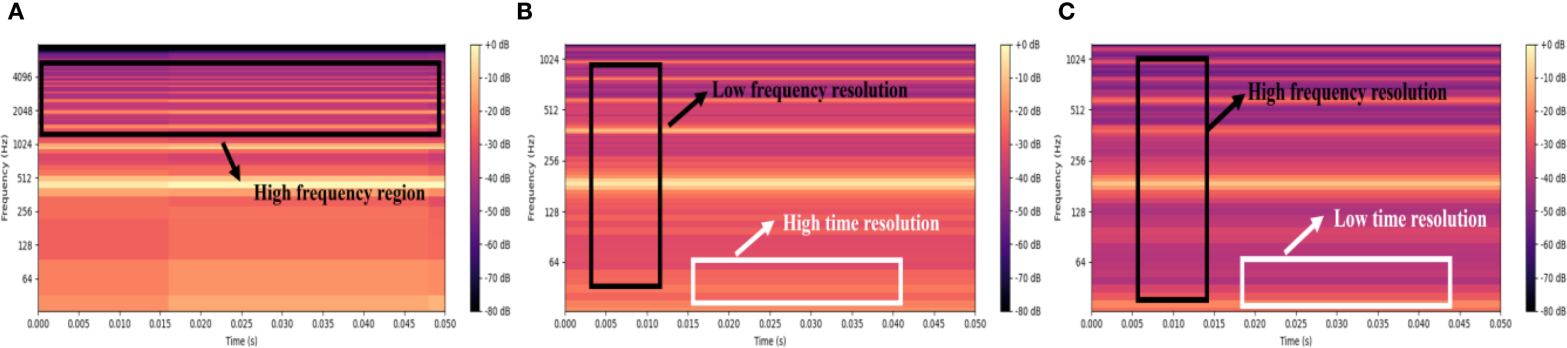
PLMS spectrograms of *Bombus terrestris* across different hyperparameters. **(A)** 16000Hz & patch size 10 **(B)** 2500Hz & patch size 10 **(C)** 2500Hz & patch size 20.

**Figure 4** illustrates a comparative analysis of PLMS spectrogram of *Bombus terrestris* acoustic signals under varying sampling rates and patch size parameters. **Figure 4A** presents the PLMS with a 16,000 Hz sampling rate and patch size of 10, covering a wide frequency range and preserving high-frequency components. However, the insect acoustic signals analyzed exhibit spectral energy predominantly concentrated in the low-frequency region, making high-frequency contributions relatively insignificant. Moreover, the high sampling rate imposes constraints on temporal resolution, leading to less detailed local feature representation in the high-frequency region. **Figure 4B** shows the PLMS with a reduced sampling rate of 2,500 Hz while maintaining a patch size of 10. This configuration decreases frequency resolution and slightly reduces the clarity of low-frequency details but significantly enhances temporal resolution, which enables more precise characterization of transient spectral dynamics and is critical for capturing short-duration acoustic events in insect signals. **Figure 4C** retains the 2,500 Hz sampling rate while increasing the patch size to 20. This adjustment markedly improves frequency resolution, producing smoother and more continuous spectral structures across the full frequency spectrum. However, the improved frequency resolution comes at the expense of temporal resolution, diminishing the ability to resolve rapid temporal variations. In summary, higher sampling rates facilitate the preservation of high-frequency spectral information but require a trade-off with temporal resolution. Larger patch sizes improve frequency resolution at the cost of temporal precision. Thus, the selection of sampling rate and patch size should be task-specific, balancing time and frequency resolution to achieve optimal and accurate feature representation.

**Figure 5:**
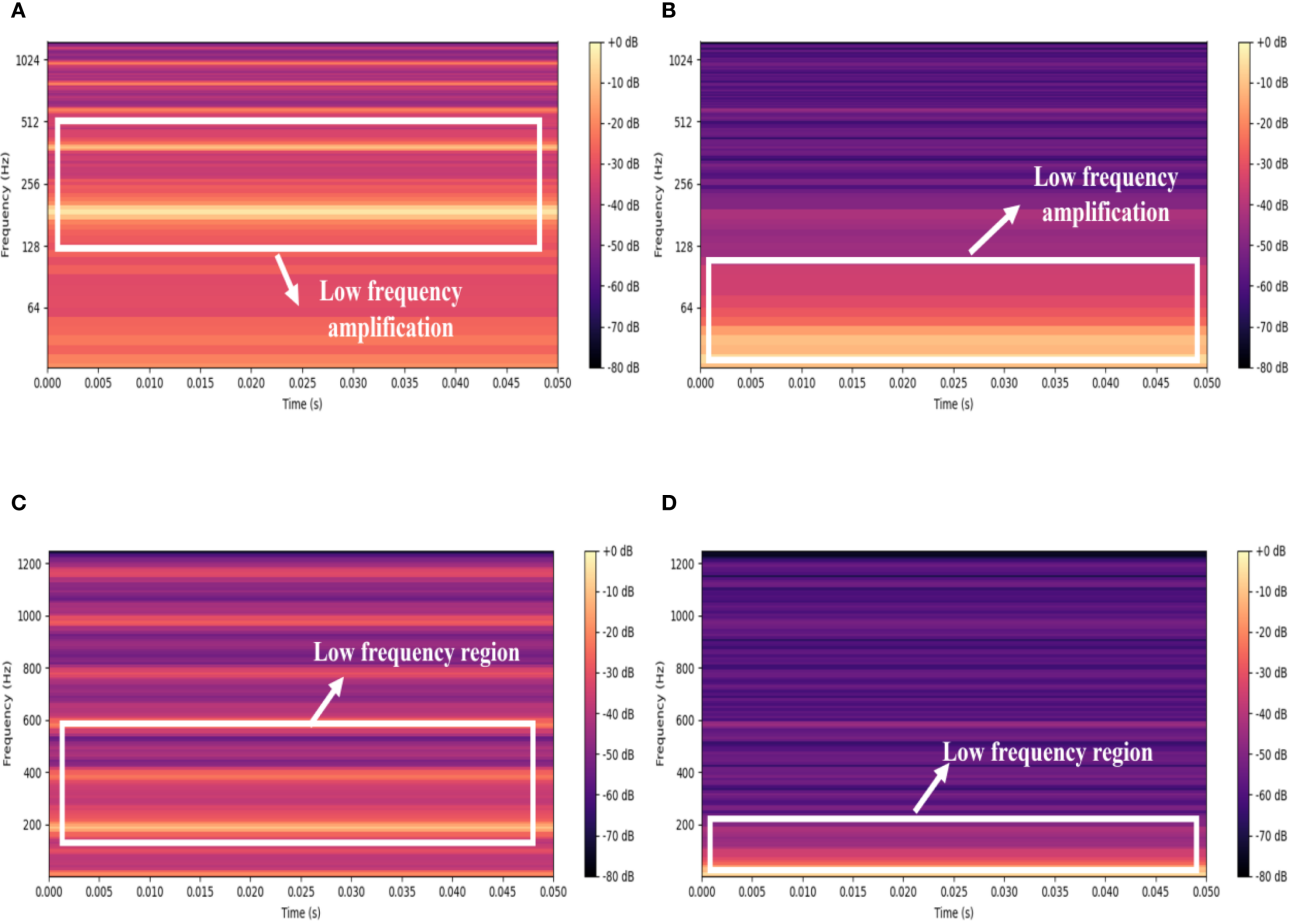
Comparison of spectrograms before and after logarithmic scaling for *Bombus terrestris* and *Bradysia difformis.* **(A)** *Bombus terrestris* with logarithmic scaling **(B)** *Bradysia difformis* with logarithmic scaling. **(C)** *Bombus terrestris* without logarithmic scaling **(D)** *Bradysia difformis* without logarithmic scaling.

**Figure 5** presents a comparison of the acoustic signal spectrograms from *Bombus terrestris* and *Bradysia difformis*, before and after logarithmic scaling. As shown in **Figures 5A, B**, the logarithmic transformation significantly amplifies and highlights the energy in the low-frequency region, effectively expanding the dynamic range in this frequency band and thereby rendering its details more discernible. In contrast, the spectrograms without logarithmic scaling in **Figures 5C, D** exhibit relatively flat low-frequency energy with a limited dynamic range, which results in the masking of low-frequency details and hinders the effective capture of subtle frequency variations. The PLMS feature, by applying logarithmic scaling, effectively compresses the influence of high-amplitude frequency components while enhancing the resolution of low-energy regions. This leads to improved robustness and representational capacity of the features, facilitating subsequent acoustic feature extraction and classification. Therefore, the logarithmic transformation of PLMS constitutes a crucial preprocessing step for enhancing the analysis of insect acoustic signals, markedly improving the expressiveness and discriminability of low-frequency components.

To summarize, the PLMS representation achieves an optimal balance between computational efficiency and feature expressiveness by integrating downsampling with logarithmic scaling. While downsampling effectively reduces computational burden, logarithmic scaling enhances the fidelity of low-frequency components that are essential for capturing nuanced bioacoustic features. Additionally, patch-level processing partitions the spectrogram into localized sub-regions, thereby amplifying transient and harmonic characteristics intrinsic to insect acoustic signals. By maintaining the inherent time-frequency continuity within these localized spectro-temporal segments, PLMS markedly improves the model’s capability to characterize the dynamic and non-stationary properties of bioacoustic signals. Collectively, these methodological innovations produce a robust and discriminative feature representation that significantly elevates classification performance across a diverse spectrum of complex bioacoustic applications.

#### 4.2.2 Baseline features

**Figure 6:**
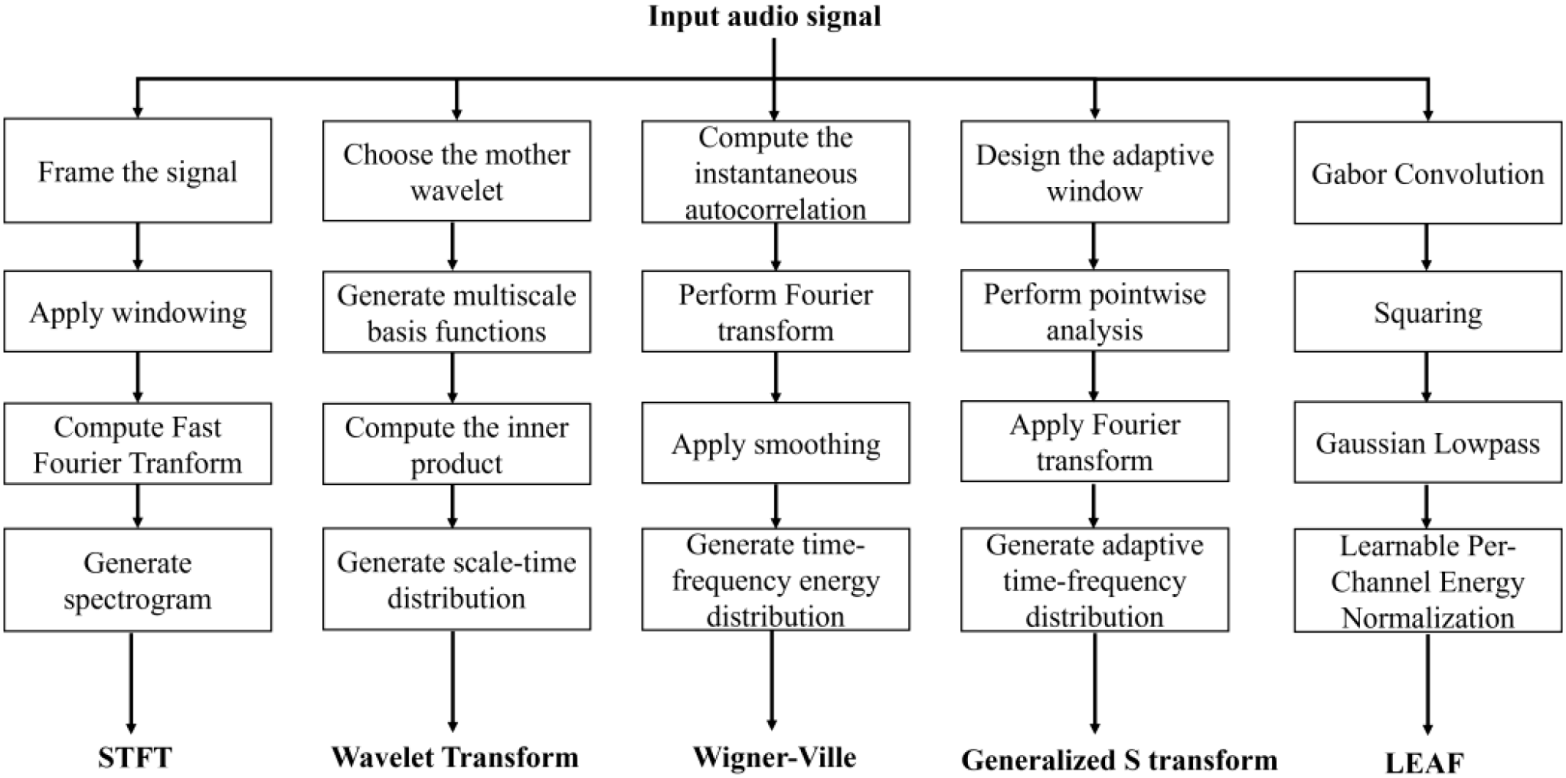
Flowchart of the feature extraction procedures for different acoustic representations.

In this study, we select five features as baseline features and juxtaposed them with the PLMS features. These five features encompass the STFT, Wavelet Transform, Wigner-Ville Distribution, Generalized S-Transform and LEAF. The extraction procedures for these features are depicted in **Figure 6**.

The STFT partitions the acoustic signal into consecutive short-time windows and applies the Fourier Transform within each window, generating the time-frequency representation. One of the key advantages of the STFT is its capability to offer localized time-frequency representations. However, the Heisenberg uncertainty principle introduces an inherent trade-off between time and frequency resolution, limiting the precision with which both can be simultaneously captured. The Wavelet Transform, in contrast, decomposes the signal using wavelet basis functions across multiple scales, enabling excellent localization in both the time and frequency domains. The ability of the Wavelet Transform to capture transient and localized variations within the signal makes it particularly effective, especially for tasks where traditional Fourier analysis fails due to its inability to resolve short-lived or time-varying features. The Wigner-Ville Distribution, a joint time-frequency analysis technique, offers high-resolution time-frequency maps, addressing the typical limitations of classical linear time-frequency methods in reconciling time and frequency resolution. However, the presence of cross-term interference can severely degrade the clarity of the time-frequency map, impairing the accuracy of the analysis. In contrast, the Generalized S-Transform provides adaptive localization by selecting an appropriate kernel function, offering greater flexibility in adjusting time and frequency resolution. Such capabilities make the method particularly well-suited for analyzing non-stationary signals with rapid and substantial variations in instantaneous frequency, which pose challenges for traditional methods that may fail to capture these dynamic characteristics. LEAF employs a learnable convolutional frontend to optimize time-frequency representations through end-to-end training. By replacing fixed filterbanks and compressors with trainable Gabor filters and adaptive per-channel energy normalization, it enables task-specific feature extraction and robust noise suppression.

#### 4.2.3 Cross-modal transfer learning with pretrained YOLO

The YOLO series of models, known for their single-stage detection architecture, have been widely adopted in computer vision due to their high-speed and accurate object detection capabilities. As a more recent iteration in this series, YOLOv11 retains the core real-time detection advantages while introducing enhanced feature extraction structures, stronger attention mechanisms, and a more lightweight design. Given the computational constraints and deployment requirements of pest acoustic classification tasks, this study adopts the YOLOv11n variant. This version maintains high accuracy while significantly reducing model parameters and computational overhead, making it well-suited for efficient inference on edge devices. Therefore, leveraging the preprocessed PLMS feature spectrograms, we employ the pre-trained YOLOv11n-cls model within a transfer learning framework and perform partial fine-tuning, updating the classification head and higher-level feature layers. This targeted adaptation preserves the generic low-level features learned during pre-training while fine-tuning the higher-level representations for the specific insect acoustics classification task, yielding an end-to-end, high-efficiency classification framework.

**Figure 7:**
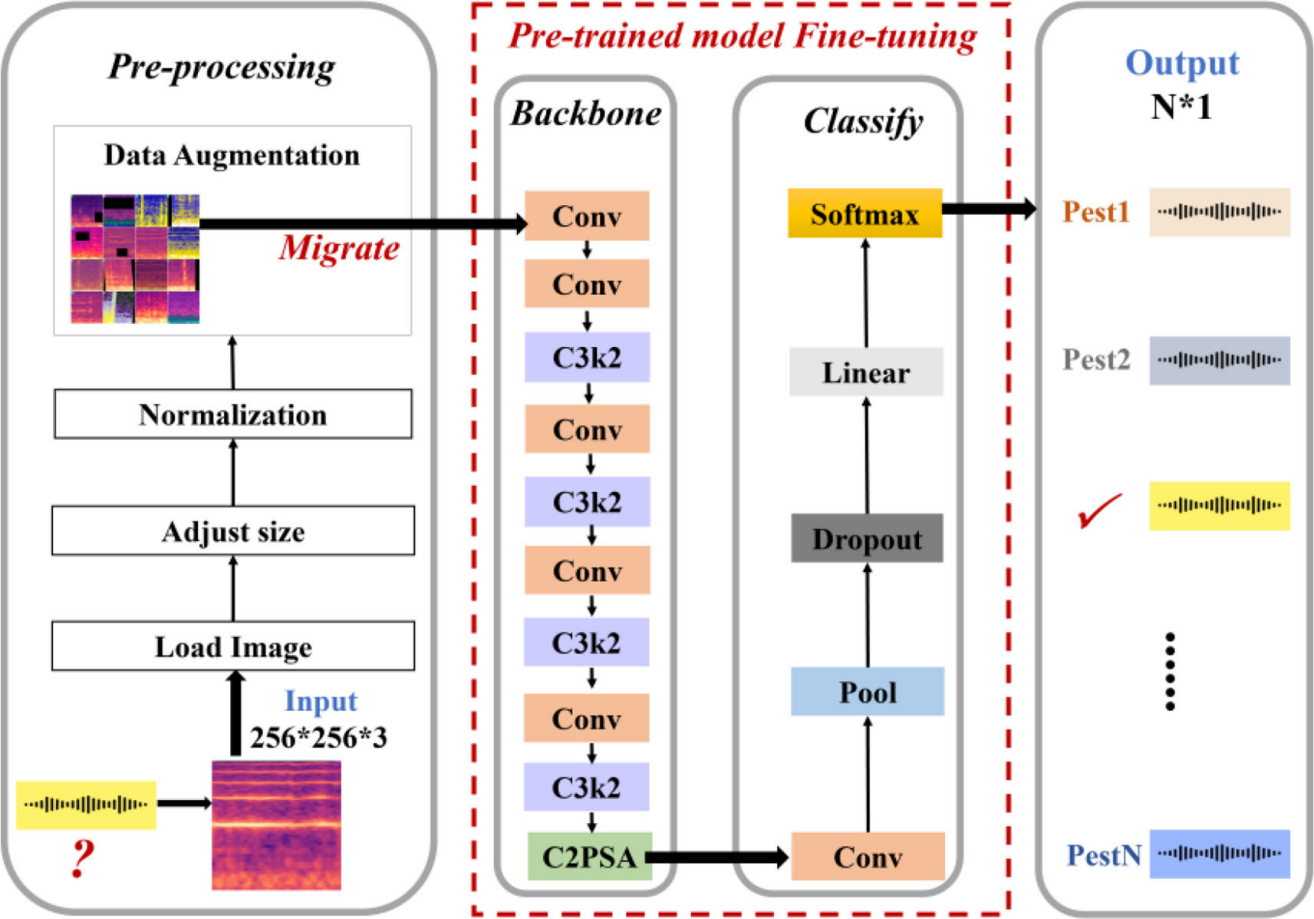
Architecture of the cross-modal transfer learning model with pretrained YOLO.

As depicted in **Figure 7**, we first optimize the input layer. The PLMS time-frequency representation of insect sounds, with dimensions 256×256×3, is preprocessed to comply with the input requirements of YOLOv11n, facilitating direct processing of multi-scale time-frequency features. Specifically, we apply resizing and normalization techniques, along with various data augmentation methods, including random cropping, random rotation, and color jittering. These strategies augment the diversity and robustness of the training dataset, thereby strengthening the model’s generalization ability and significantly enhancing its accuracy in capturing the intrinsic variability of insect sound patterns.

Next, the augmented data is fed into the Backbone layer of the pre-trained model. We refine the Backbone architecture by introducing the C3 module with kernel size 2 (C3k2) structure, replacing several conventional convolutional layers. The C3k2 module represents a deep optimization of the traditional Cross Stage Partial Network (CSP) Bottleneck structure, aiming to improve feature extraction efficiency through parallel convolution designs and flexible parameter configurations. This module divides the input feature map into two branches: one is passed directly through to preserve shallow details, while the other undergoes multi-scale feature extraction via the C3k module, which employs variable convolution kernels, such as 3×3 or 5×5. The extracted features are then concatenated and fused. This design not only captures subtle high-frequency vibrations but also suppresses low-frequency environmental noise, ensuring a robust representation of acoustic features. Furthermore, this approach reduces redundant computations, accelerates inference speed, and employs grouped convolutions and channel compression for lightweight optimization. These enhancements make the model particularly well-suited for deployment on IoT edge devices in agricultural fields, offering high precision with minimal resource consumption. Additionally, we integrate the Cross Stage Partial with Pyramid Squeeze Attention (C2PSA) module into the Backbone. Based on the CSP structure, this module segments feature processing and incorporates the Pyramid Slice Attention mechanism to dynamically adjust spatial attention. Through multi-scale convolution kernels and channel weighting, the module significantly enhances the expression of the PLMS time-frequency dynamics, thereby improving the model’s sensitivity to the frequency patterns of pest activities.

Finally, during the design of the classification module, we adopt a hybrid approach that combines global feature extraction with classification output, while freezing the detection head parameters to facilitate the expansion of classification-derived tasks. This module comprises convolutional layers, pooling layers, Dropout layers, linear layers, and Softmax layers: the convolutional layers extract high-dimensional features, the pooling layers perform downsampling to reduce dimensionality, the Dropout layers prevent overfitting, the linear layers map the features to the class space, and the Softmax layer outputs the class probabilities. This design strikes an optimal balance between computational efficiency and classification accuracy, making it well-suited for real-time classification tasks in complex environments. Consequently, the model proposed in this paper can effectively extract deep semantic information from PLMS, offering significant advantages over traditional methods in terms of parameter count, computational cost, and deployment efficiency.

The detailed parameter configuration of the proposed model is presented in **Table 4**. This model comprises 11 sequential stages, integrating five convolutional layers (Conv), three C3K2 modules, and one C2PSA module to enhance feature extraction efficiency while maintaining an effective trade-off between accuracy and computational complexity. The backbone begins with two Conv layers utilizing 3×3 kernels with a stride of 2 to extract low-level features while reducing spatial dimensions. To enhance feature extraction, the C3K2 module is introduced in Stage 3, incorporating multi-scale convolutional kernels, though without residual connections, and applying a channel reduction ratio of 0.25 to optimize efficiency. Stage 4 follows with another Conv layer, further refining feature maps, while Stage 5 reintroduces the C3K2 module. As the model progresses, Stage 6 applies a Conv layer, followed by Stage 7, where a C3K2 module integrates residual connections to improve gradient flow and feature learning. Stage 8 continues with Conv processing, while Stage 9 employs another residual-connected C3K2 module to maintain deeper feature representations. The C2PSA module in Stage 10 enhances spatial attention through pyramid slice attention mechanisms, refining classification performance. Finally, Stage 11 serves as the classification layer, reducing feature maps to 12 output categories corresponding to different pest species. This architecture is designed to efficiently extract multi-scale features while maintaining robust classification performance for pest detection.

**Table 4:** The detailed parameter configuration of our model.

Stage	Operator	Filter	Kernel	Stride	Residual connection	Channel reduction ratio
1	Conv	16	3×3	2	/	/
2	Conv	32	3×3	2	/	/
3	C3K2	64	/	/	×	0.25
4	Conv	64	3×3	2	/	/
5	C3K2	128	/	/	×	0.25
6	Conv	128	3×3	2	/	/
7	C3K2	128	/	/	✓	/
8	Conv	256	3×3	2	/	/
9	C3K2	256	/	/	✓	/
10	C2PSA	256	/	/	/	/
11	Classify	12	/	/	/	/

## 5 Additional requirements

### 5.1 Implementation details

In this study, all models were implemented in the PyTorch DL framework and executed on the workstation with a 12th Gen Intel^®^ Core™ i7-12700F Processor (2.10 GHz), 32.0 GB RAM, and one NVIDIA GeForce RTX 4070 GPU.

During the preprocessing stage, the original sampling rate of the acoustic signal is 16KHz. The cutoff frequency is set to half the sampling rate. Window function selects hamming window. The order of the filter is set to 100. Moreover, the training and testing datasets are split in a ratio of 8:2. Additionally, multiple rounds of 5-fold cross-validation are performed to further assess the effectiveness and robustness of the proposed algorithm across different data partitions. The image size of the input model network is 256*256. The models are trained for 150 epochs on each mini-batch with a batch size of 32. The loss function used in all experiments is cross-entropy. All compared models apply early stopping with a patience of five epochs, whereas our method is trained without early stopping. The hyperparameter settings are as follows: ResNet18 and DenseNet adopt the Adam optimizer, whereas EfficientNet-B0, VGG19, MobileNet, and our proposed model employ SGD. The learning rates are set to 0.001 for ResNet18, DenseNet, and MobileNet; 0.01 for EfficientNet-B0 and VGG19; and 0.1 for our model. Dropout is applied at rates of 0.2 for ResNet18, EfficientNet-B0, and MobileNet, and 0.5 for VGG19. DenseNet and our proposed method do not utilize dropout.

### 5.2 Evaluation

In this study, several evaluation metrics are employed to assess model performance. The confusion matrix analyzes the true versus predicted classifications, providing insight into the types of errors made by the model through the enumeration of true positives, true negatives, false positives and false negatives. Top-1 accuracy (Accuracy@1) measures the proportion of instances where the top predicted class matches the true class, reflecting primary classification accuracy. Macro-Recall quantifies the ratio of true positives to the total number of actual positive instances for each class, then takes the arithmetic mean across all classes. This approach ensures equal evaluation weight for all categories, which is critical when each class holds independent importance. The Macro-F1 score, the harmonic mean of Macro-Precision and Macro-Recall, offers a balanced metric that accounts for the trade-off between these two measures, adopting macro-averaging to ensure uniform assessment of classification consistency. Lastly, the receiver operating characteristic (ROC) curve provides a graphical representation of discriminative power across various classification thresholds, plotting the true positive rate against the false positive rate. The Macro-area under the ROC curve (AUC) serves as an aggregate measure of performance calculated by macro-averaging AUC values across classes, with higher AUC values indicating superior classification ability in maintaining inter-class decision boundary coherence. We deliberately employ macro-averaging to guarantee metric interpretability from a class-agnostic perspective, as this method equally weights the decision patterns of all categories. Furthermore, the number of parameters (in millions) and the computational complexity in GigaFloating Point Operations Per Second (GFLOPS) are used in this study. The number of parameters in the model represents the model’s size and capacity, and GFLOPS is a measure of the computational complexity of the model, with higher values indicating more computation is required.

## 6 Results & analysis

In this section, we present the results of classification validation using six models, namely the model used in this paper, Resnet18 (He et al., 2016) (He Ket al., 2016), Efficientnet-b0 (Tan and Le, 2019) (Tan M et al., 2019), VGG19 (Simonyan and Zisserman, 2014) (Simonyan and Zisserman, 2014), MobileNetV2 (Sandler et al., 2018) (Sandler et al., 2018), DenseNet (Huang et al., 2017) (Huang G et al., 2017), for different features extracted from the InsectSound1000 dataset. Additionally, we conducted ablation studies and comparisons with other algorithms to further validate the effectiveness and robustness of our proposed method.

### 6.1 Comparative analysis of feature dimensionality

To ensure rigorous benchmarking, the experimental parameters are standardized across all models: the parameter *SegmentFrameNumber Num* is fixed at 10 frames per audio segment and the *samplingrate* is set to 16KHz. As shown in **Table 4**, this study demonstrates the superior performance of the proposed PLMS feature and the corresponding model (denoted as Ours in **Table 5**) through extensive comparative experiments. Under the PLMS feature representation, our model achieves the highest performance, attaining an Accuracy@1 of 92.4%. Additionally, it achieves a Macro-AUC of 99.70%, significantly surpassing the performance of other models. This underscores the strong discriminative capability of the PLMS feature, which is further leveraged by our model through tailored network design. The superiority of the PLMS feature becomes even more apparent when compared to other feature representations. For instance, within our model, the Accuracy@1 achieved with the PLMS feature is 92.42%, which is 1.61%, 4.34%, 8.5%, 10.13% and 48.97% higher than those achieved with the S-Transform, STFT, wavelet-based feature, Wigner-Ville and LEAF, respectively, with accuracies of 90.96%, 88.58%, 83.92%, 84.92% and 62.04%. This performance improvement can be attributed to the ability of the PLMS feature to integrate the temporal dynamics of pest acoustic signals with multi-scale frequency domain representations, enabling a more comprehensive characterization of audio semantic information. In contrast, baseline features such as S-Transform, STFT, wavelet, and Wigner-Ville predominantly focus on either time-domain or frequency-domain information in isolation. This singular focus limits their capacity to fully capture the complex and multi-faceted nature of audio signals, thereby constraining their generalization capabilities. For instance, S-Transform and STFT primarily emphasize frequency-domain information, while wavelet and Wigner-Ville focus more on time-frequency representations but may not capture the multi-scale characteristics as effectively as the PLMS feature. Although LEAF employs a learnable frontend to adaptively optimize time-frequency representations and enhance robustness, it may still be less effective than PLMS at fully capturing temporal dynamics across multiple scales.

**Table 5:** Comparison of classification performance across different audio features and model combinations.

Features	Models	Accuracy@1	Macro-recall	Macro-F1	Macro-AUC
PLMS	Ours	**92.42**	**92.42**	**92.41**	**99.70**
	Resnet18	87.59	87.59	87.53	97.93
	Efficientnet-b0	88.45	88.45	88.44	99.28
	VGG19	8.33	8.33	1.28	50.00
	Mobilenet	84.26	84.26	84.48	98.55
	DenseNet	77.34	77.34	77.42	97.31
S-Transform	Ours	90.96	90.96	90.99	99.55
	Resnet18	73.61	73.61	73.71	94.66
	Efficientnet-b0	83.87	83.87	83.82	98.73
	VGG19	41.58	41.58	41.70	79.48
	Mobilenet	60.97	61.00	61.57	90.88
	DenseNet	9.22	9.22	2.45	72.71
STFT	Ours	88.58	88.58	88.63	99.33
	Resnet18	73.36	73.36	73.13	94.78
	Efficientnet-b0	81.92	81.92	81.91	98.48
	VGG19	44.94	44.94	43.97	82.73
	Mobilenet	62.55	62.55	61.10	92.57
	DenseNet	9.19	9.19	2.32	65.90
Wavelet	Ours	83.92	83.92	83.99	98.69
	Resnet18	60.65	60.65	60.18	90.09
	Efficientnet-b0	78.37	78.37	78.34	97.94
	VGG19	39.38	39.38	39.39	78.06
	Mobilenet	55.97	55.97	55.65	89.05
	DenseNet	8.55	8.55	1.74	67.51
Wigner_ Ville	Ours	84.92	84.92	84.93	98.94
	Resnet18	68.47	68.47	68.47	93.04
	Efficientnet-b0	78.89	78.89	78.79	97.94
	VGG19	43.50	43.50	42.60	81.59
	Mobilenet	57.40	57.41	57.02	90.15
	DenseNet	8.33	8.33	1.28	52.30
LEAF	Ours	62.04	62.04	61.62	93.14
	Resnet18	62.25	62.25	62.28	90.25
	Efficientnet-b0	60.12	60.12	59.89	92.58
	VGG19	40.75	40.75	40.31	79.90
	Mobilenet	54.32	53.46	53.27	89.13
	DenseNet	42.88	42.88	43.34	82.76

Bold values indicate the highest performance achieved by the proposed algorithm among all compared methods for each metric, including Accuracy@1, Macro-recall, Macro-F1, and Macro-AUC.

From the perspective of model comparison, our model demonstrates a comprehensive advantage under the PLMS feature, achieving an Accuracy@1 of 92.42%, a Macro-F1 score of 92.41%, and a Macro-AUC of 99.70%, significantly outperforming other models. In contrast, ResNet18, although yielding the second-best performance under the PLMS feature with an Accuracy@1 of 87.59%, still trails our model by 4.83%. While EfficientNet-b0 benefits from a compound scaling strategy that enhances computational efficiency, its Accuracy@1 is 3.97% lower than that of our model, highlighting the superior capability of our model to capture long-range temporal dependencies and integrate cross-scale features.

It is noteworthy that traditional deep networks exhibit significant sensitivity to feature representations. For example, VGG19 achieves an Accuracy@1 of only 8.33% and a Macro-F1 of 1.28% under the PLMS feature. This poor performance is largely attributed to its fixed receptive field and redundant parameter design, which are ill-suited for capturing dynamic audio features. DenseNet achieves an Accuracy@1 of only 9.22% under the S-Transform feature, reflecting the limitations of its dense connectivity mechanism in capturing frequency-domain discontinuities. In contrast, MobileNet performs reasonably well with the PLMS feature, achieving an Accuracy@1 of 84.26%. However, its lightweight design compromises its ability to extract deep semantic features, as evidenced by its Accuracy@1 of only 60.97% under the S-Transform feature, considerably lower than the 90.96% achieved by our model. This suggests that, although depthwise separable convolutions are employed in MobileNet to reduce computational overhead, the model’s capacity to capture complex acoustic patterns remains limited. Collectively, these findings underscore the importance of the coordinated optimization of the PLMS feature representation and the architectural design of our model as the key determinant of performance improvements.

### 6.2 Comparative analysis of various models

As presented in **Table 6**, the number of parameters (in millions) and the computational complexity in GFLOPS of various models under the PLMS feature are systematically compared. From the perspective of parameter quantity, VGG19 has the highest number of parameters, reaching 139.62 million. This substantial parameter volume is a direct consequence of its deep network architecture, making it well-suited for high-precision applications that are less constrained by computational resources. In contrast, ResNet18 contains 11.23 million parameters, while DenseNet has 6.85 million. ResNet18 balances depth and efficiency through residual connections, whereas DenseNet enhances feature reuse via densely connected layers, though at the expense of a larger number of parameters compared to lightweight models. EfficientNet-b0 further reduces parameters to 4.02 million by optimizing parameter utilization through a compound scaling strategy. MobileNet compresses parameters even further to 2.24 million by employing depthwise separable convolutions, showcasing the advantages of lightweight network design. Notably, our model achieves the most aggressive parameter reduction, with only 1.54 million parameters, even surpassing MobileNet. This result underscores its structural innovations in pre-trained model design and fine-tuning strategies.

**Table 6:** Comparative analysis of model parameters and computational efficiency under the PLMS feature.

Feature	Models	Param (million)	GFLOPS (GigaFLOPs)
PLMS	Ours	1.54M	0.01
	Resnet18 (He et al., 2016)	11.23M	0.07
	Efficientnet-b0 (Tan and Le, 2019)	4.02M	0.02
	VGG19 (Simonyan and Zisserman, 2014)	139.62M	1.04
	Mobilenet (Sandler et al., 2018)	2.24M	0.01
	DenseNet (Huang et al., 2017)	6.85M	0.11

Regarding computational complexity, VGG19 incurs the highest computational cost, with a GFLOPS of 1.04, primarily due to its extensive fully connected layers and deep convolutional operations. ResNet18 and DenseNet maintain moderate computational complexity, with GFLOPS values of 0.07 and 0.11, respectively. While their computational demands are consistent with their parameter volumes, DenseNet incurs additional computational overhead due to its dense connectivity pattern. EfficientNet-b0 significantly reduces computational demands to 0.02 through a well-balanced scaling mechanism. MobileNet and our model achieve the lowest computational complexity, with a GFLOPS of 0.01, highlighting their superior efficiency. This characteristic makes them particularly suitable for real-time processing and low-power environments. In summary, our model emerges as the most lightweight architecture in the comparison, with both the lowest parameter count of 1.54 million and the lowest GFLOPS of 0.01. These results emphasize its advantages in model compression and computational optimization, rendering it highly applicable to scenarios with stringent resource constraints.

### 6.3 Comparative analysis of sampling rate dimensionality

To ensure rigorous benchmarking, the experimental parameters *SegmentFrameNumber Num* is fixed at 10 frames per audio segment. The original sampling rate is 16KHz. As shown in **Table 7**, we compare classification performance across different sampling rates and model combinations based on the PLMS feature. On one hand, a comprehensive analysis of the dynamic correlation between model performance and sampling rate reveals significant variations among different architectures. Under the PLMS feature, our model exhibits optimal performance at 2,500 Hz, achieving an Accuracy@1 of 96.49%, a Macro-F1 score of 96.49%, and a Macro-AUC of 99.93%. In contrast, VGG19 demonstrates extreme instability at higher sampling rates, particularly at 16 kHz, where its Accuracy@1 plunges to 8.33%, and Macro-F1 drops to 1.28%. This can be attributed to the densely connected fully connected layers in VGG19, which amplify sensitivity to high-resolution noise artifacts. Consequently, the model tends to overfit specific frequency bands, leading to a pronounced degradation in generalization performance. While ResNet18 and EfficientNet-b0 exhibit relatively stable performance across different sampling rates, their performance still shows a slight degradation as the sampling rate decreases. ResNet18 achieves peak performance at 1,500 Hz, attaining an Accuracy@1 of 93.78%, while EfficientNet-b0 performs optimally at 3,000 Hz and 4,000 Hz, reaching an Accuracy@1 of 93.32%. These differences arise from the architectural characteristics of ResNet18 and EfficientNet-b0. ResNet18 utilizes residual connections to effectively mitigate gradient vanishing, enhancing its adaptability to multi-sampling-rate features. In contrast, EfficientNet-b0 employs neural architecture search to optimize the balance between depth, width, and resolution, thereby improving computational efficiency across various sampling rates.

**Table 7:** Comparison of classification performance across different sample rates and model combinations based on the PLMS feature.

Sampling rate (Hz)	Models	Accuracy@1	Macro-recall	Macro-F1	Macro-AUC
Original	Ours	92.42	92.42	92.41	99.70
	Resnet18	87.59	87.59	87.53	97.93
	Efficientnet-b0	88.45	88.45	88.44	99.28
	VGG19	8.33	8.33	1.28	50.00
	Mobilenet	84.26	84.26	84.48	98.55
	DenseNet	77.34	77.34	77.42	97.31
4000	Ours	95.29	95.29	95.29	99.86
	Resnet18	92.26	92.26	92.26	99.26
	Efficientnet-b0	93.32	93.32	93.33	99.57
	VGG19	89.55	89.55	89.60	98.99
	Mobilenet	90.74	90.74	90.74	99.30
	DenseNet	89.88	89.88	89.92	99.24
3500	Ours	96.03	96.03	96.02	99.87
	Resnet18	91.60	91.60	91.68	99.22
	Efficientnet-b0	92.20	92.20	92.17	99.45
	VGG19	90.48	90.48	90.46	99.25
	Mobilenet	90.01	90.08	90.03	99.21
	DenseNet	91.20	91.20	91.17	99.40
3000	Ours	95.63	95.63	95.62	99.85
	Resnet18	93.25	93.25	93.27	99.66
	Efficientnet-b0	93.32	93.32	93.28	99.56
	VGG19	91.70	91.07	91.02	99.31
	Mobilenet	88.82	88.82	88.81	99.22
	DenseNet	89.95	89.95	89.89	99.44
2500	Ours	**96.49**	**96.49**	**96.49**	**99.93**
	Resnet18	92.86	92.86	92.84	99.42
	Efficientnet-b0	92.00	92.00	92.01	99.59
	VGG19	90.61	90.61	90.61	99.34
	Mobilenet	86.51	86.57	86.81	98.61
	DenseNet	92.13	92.13	92.11	99.44
2000	Ours	95.21	95.16	95.15	99.82
	Resnet18	93.12	93.12	93.13	99.43
	Efficientnet-b0	92.33	92.33	92.31	99.54
	VGG19	91.01	91.01	91.00	99.29
	Mobilenet	91.67	91.67	91.73	99.51
	DenseNet	89.42	89.42	89.55	99.21
1500	Ours	96.16	96.16	96.17	99.92
	Resnet18	93.78	93.78	93.76	99.56
	Efficientnet-b0	92.92	92.92	92.90	99.60
	VGG19	89.81	89.81	89.79	99.19
	Mobilenet	88.96	88.96	88.92	99.20
	DenseNet	91.80	91.80	91.76	99.29
1000	Ours	95.44	95.44	95.45	99.85
	Resnet18	93.58	93.58	93.57	99.40
	Efficientnet-b0	92.53	92.53	92.49	99.56
	VGG19	89.29	89.29	89.23	99.07
	Mobilenet	91.53	91.53	91.51	99.63
	DenseNet	90.81	90.81	90.84	99.30

Bold values indicate the highest performance achieved by the proposed algorithm among all compared methods for each metric, including Accuracy@1, Macro-recall, Macro-F1, and Macro-AUC.

In contrast, lightweight models such as MobileNet and DenseNet perform reasonably well at mid-to-low sampling rates but degrade significantly at the original high sampling rate. MobileNet achieves its best Accuracy@1 (91.67%) at 2,000 Hz, while DenseNet peaks at 2,500 Hz with an Accuracy@1 of 92.13%. However, at 16 kHz, the Accuracy@1 of MobileNet drops to 84.26%, while DenseNet declines sharply to 77.34%. This performance gap stems from their distinct architectural constraints. MobileNet employs depthwise separable convolutions, which effectively reduce the number of parameters but also weaken cross-channel dependencies, resulting in suboptimal performance on high-resolution data. In contrast, DenseNet faces increased computational complexity at high sampling rates, where excessive redundant features introduce gradient noise accumulation during backpropagation, ultimately impairing model convergence stability. Notably, despite being designed as a lightweight model, our model demonstrates remarkable cross-sampling-rate stability. By integrating structural innovations, it effectively compensates for the information loss induced by downsampling, thereby enhancing classification robustness under low-sampling-rate conditions. This suggests that lightweight architectures, when appropriately designed, can mitigate performance degradation across varying sampling rates.

One the other hand, examining metric consistency and model reliability further highlights the impact of sampling rates. All models exhibit Macro-AUC values consistently exceeding 99%, indicating strong class discrimination capability. However, discrepancies between Macro-F1 and Accuracy@1 reveal subtle classification biases. For instance, VGG19 achieves a Macro-AUC of 99.29% at 2,000 Hz, yet its Macro-F1 remains lower at 91.00%, suggesting potential class imbalance issues or suboptimal recognition of minority classes. In contrast, our model maintains exceptional consistency across all sampling rates, with Accuracy@1, Macro-F1, and Macro-AUC remaining highly aligned. At 2,500 Hz, it achieves an Accuracy@1 of 96.49%, a Macro-F1 of 96.49%, and a Macro-AUC of 99.93%, confirming its balanced classification capability and low-variance characteristics. These findings underscore that sampling rate has a profound impact on model performance, necessitating a delicate balance between lightweight design and data adaptability. The results suggest that while certain architectures, such as VGG19, struggle with high-resolution noise sensitivity, others, like ResNet18 and EfficientNet-b0, exhibit better adaptability to varying sampling rates. More importantly, our model, through architectural innovation, achieves robust performance at lower sampling rates, making it a highly efficient and practical solution for real-world deployment, particularly in resource-constrained environments where computational efficiency and robustness are crucial.

### 6.4 Comparative analysis of patch size

To rigorously analyze the impact of varying patch sizes on the classification performance of different models based on the PLMS, the downsampling rate is set to 2500Hz, as indicated by the results in **Table 7**. Subsequently, as delineated in **Table 8**, we conducted a comparative analysis across four patch sizes. Experimental results demonstrate that patch size has a significant impact on model performance. The proposed model achieves optimal performance when the patch size is configured to a temporal window encompassing 10 successive frames, attaining an Accuracy@1 of 96.49%, a Macro-F1 and Macro-Recall of 96.49%, and a Macro-AUC as high as 99.93%, significantly outperforming the other four models. This finding suggests that the patch size of 10 frames effectively balances local detail and global contextual information. In contrast, a patch size of 5 frames may lack sufficient temporal correlation, leading to incomplete feature representations, while patch sizes of 15 or 20 frames could introduce redundant noise, diminishing the model’s sensitivity to informative signals.

**Table 8:** Comparison of classification performance of different combinations of patch sizes and models based on the PLMS feature.

Patch size (segmentframenumber)	Models	Accuracy@1	Macro-recall	Macro-F1	Macro-AUC
5	Ours	94.44	94.44	94.43	99.83
	Resnet18	93.39	93.39	93.40	99.57
	Efficientnet-b0	92.72	92.72	92.74	99.52
	VGG19	90.34	90.34	90.35	99.24
	Mobilenet	87.86	87.10	87.15	99.08
	DenseNet	90.94	90.94	90.90	99.27
10	Ours	**96.49**	**96.49**	**96.49**	**99.93**
	Resnet18	92.86	92.86	92.84	99.42
	Efficientnet-b0	92.00	92.00	92.01	99.59
	VGG19	90.61	90.61	90.61	99.34
	Mobilenet	86.51	86.57	86.81	98.61
	DenseNet	92.13	92.13	92.11	99.44
15	Ours	95.50	95.50	95.51	99.84
	Resnet18	93.65	93.65	93.68	99.54
	Efficientnet-b0	92.33	92.33	92.29	94.48
	VGG19	90.41	90.41	90.41	99.36
	Mobilenet	89.04	88.89	88.81	99.27
	DenseNet	92.26	92.26	92.29	99.59
20	Ours	93.98	93.98	93.97	99.79
	Resnet18	91.34	91.34	91.38	99.25
	Efficientnet-b0	91.01	91.01	90.99	99.34
	VGG19	90.61	90.61	90.60	99.20
	Mobilenet	90.02	89.15	89.36	99.10
	DenseNet	89.35	89.35	89.33	99.38

Bold values indicate the highest performance achieved by the proposed algorithm among all compared methods for each metric, including Accuracy@1, Macro-recall, Macro-F1, and Macro-AUC.

From the perspective of model-specific characteristics, ResNet18 attains an Accuracy@1 of 92.86% when the patch size is 10 but experiences a decline to 91.34% at a patch size of 20. This decrease arises from interference due to redundant information, underscoring the residual network’s proclivity for localized temporal contexts. EfficientNet-b0 maintains relatively stable Accuracy@1 across different patch sizes (ranging from 91.01% to 92.72%). However, when the patch size is 15, it exhibits an anomalous drop in Macro-AUC to 94.48%, which results in spectral confusion among certain categories within the PLMS features. VGG19 exhibits minimal sensitivity to patch size variations, maintaining an Accuracy@1 between 90.34% and 90.61%, as its fixed receptive field and extensive parameterization limit its capacity for capturing dynamic temporal features. MobileNet performs the worst when a patch size is 10 (an Accuracy@1 reaching only 86.51%) but recovers to 89.04% at a patch size of 15. This suggests that its lightweight architecture struggles with modeling medium-length sequences, while longer sequences partially mitigate this limitation through increased information density. DenseNet achieves a peak Accuracy@1 of 92.26% at a patch size of 15 but drops to 89.35% when the patch size reaches 20, likely due to gradient redundancy or noise propagation within its densely connected structure under long-sequence conditions.

In terms of classification metric consistency, the Macro-Recall and Macro-F1 values of all models are highly similar, indicating good inter-class balance in classification results. However, MobileNet exhibits a slight discrepancy at a patch size of 20, with a Macro-Recall of 89.15%, marginally lower than its Macro-F1 score of 89.36%. This suggests reduced sensitivity to certain low-frequency or low-amplitude categories. Notably, EfficientNet-b0 exhibits an anomalous performance at a patch size of 15, with its Macro-AUC decreasing sharply to 94.48%, significantly deviating from the consistently higher Macro-AUC values observed at other patch sizes. This decline suggests that, at this specific patch size, spectral feature confusion occurs among certain categories within longer sequences, thereby impairing the model’s discriminative capabilities. Such observations highlight the necessity of jointly modeling local and global features in temporal signal processing to effectively capture both detailed and contextual information.

### 6.5 Comparative analysis of confusion matrix and training convergence

**Figure 8:**
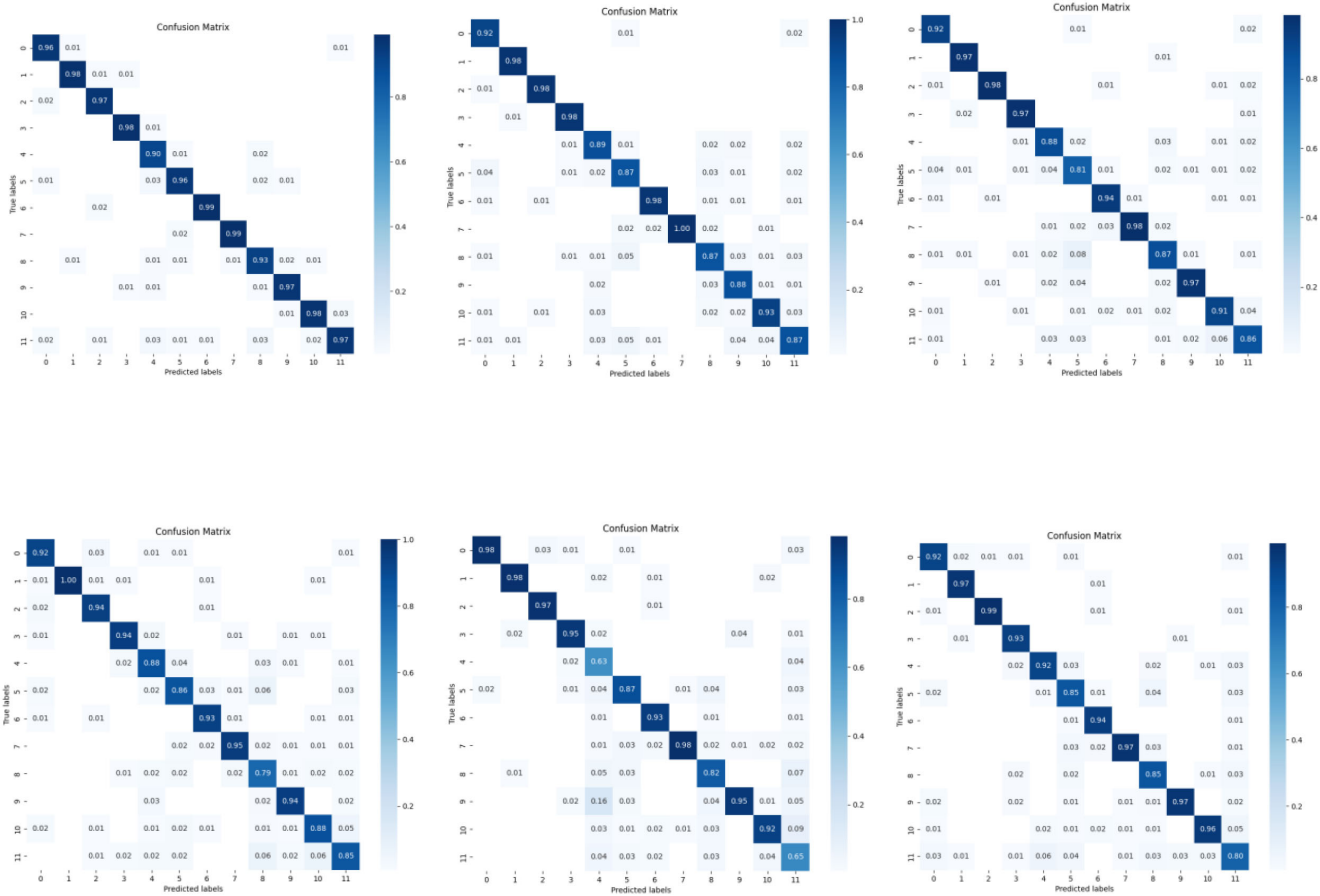
Confusion matrices of the six models based on the PLMS feature.

**Figure 8** shows the confusion matrices of 12 insect species for the six models based on the PLMS feature, with a patch size of 20 and a downsampling rate of 2,500 Hz. The horizontal axis represents the predicted labels, while the vertical axis represents the true labels, with 0–11 corresponding to the insect species listed in **Table 3** above. It is worth mentioning that our model exhibits remarkable superiority, as evidenced by its confusion matrix. Except for the 4^th^ and 8^th^ classes, where the diagonal elements are relatively low, all other classes have diagonal elements approaching 1.0, indicating exceptionally high classification accuracy. Moreover, the off-diagonal elements are minimal, signifying an exceedingly low misclassification rate. In contrast, models such as ResNet18, EfficientNet-B0, VGG19, MobileNet, and DenseNet, while achieving high accuracy in certain categories, exhibit significant misclassifications in specific classes. For instance, ResNet18 shows higher off-diagonal values for the 6^th^, 8^th^, 9^th^, and 11^th^ classes; EfficientNet-B0 for the 4^th^, 5^th^, 8^th^, and 11^th^ classes; VGG19 for the 4^th^, 5^th^, 8^th^, 10^th^, and 11^th^ classes; and DenseNet for the 5^th^, 8^th^, and 11^th^ classes. Notably, MobileNet exhibits the diagonal values of approximately 0.6 for the 4^th^ and 11^th^ classes.

Focusing on our experimental results, the confusion matrix reveals significant misclassification issues between the 4^th^ and 8^th^ classes. Samples of the 4^th^ class are primarily misclassified as the 5^th^ and 11^th^ classes, each accounting for 3%, while samples of the 8^th^ class are misclassified as the 4^th^ and 5^th^ classes, each accounting for 2%, and as the 11^th^ class, accounting for 3%. Since the data are collected under controlled conditions with minimal background noise, and based on our careful inspection of the spectrograms, the spectral features of the 4^th^, 5^th^, 8^th^, and 11^th^ classes exhibit minimal differences. Their frequency distributions and energy concentration regions highly overlap, making traditional spectrogram-based features insufficient to effectively distinguish these classes. Additionally, although the training data quality is high, the diversity of sound samples may be inadequate to capture subtle acoustic variations present in real-world conditions, which limits the model’s discriminative power.

To address these challenges, we propose several improvements. First, incorporate richer and more discriminative acoustic features such as transient signal characteristics or nonlinear dynamic features. Second, exploring additional data augmentation techniques, such as pitch shifting and the introduction of simulated environmental effects, may further enhance the diversity of insect acoustic samples and improve the model’s generalization ability. Finally, consider multimodal fusion approaches by integrating additional sensory data such as insect vibration signals and behavioral patterns to better differentiate similar classes and reduce misclassification rates between the 4^th^ and 8^th^ classes.

**Figure 9:**
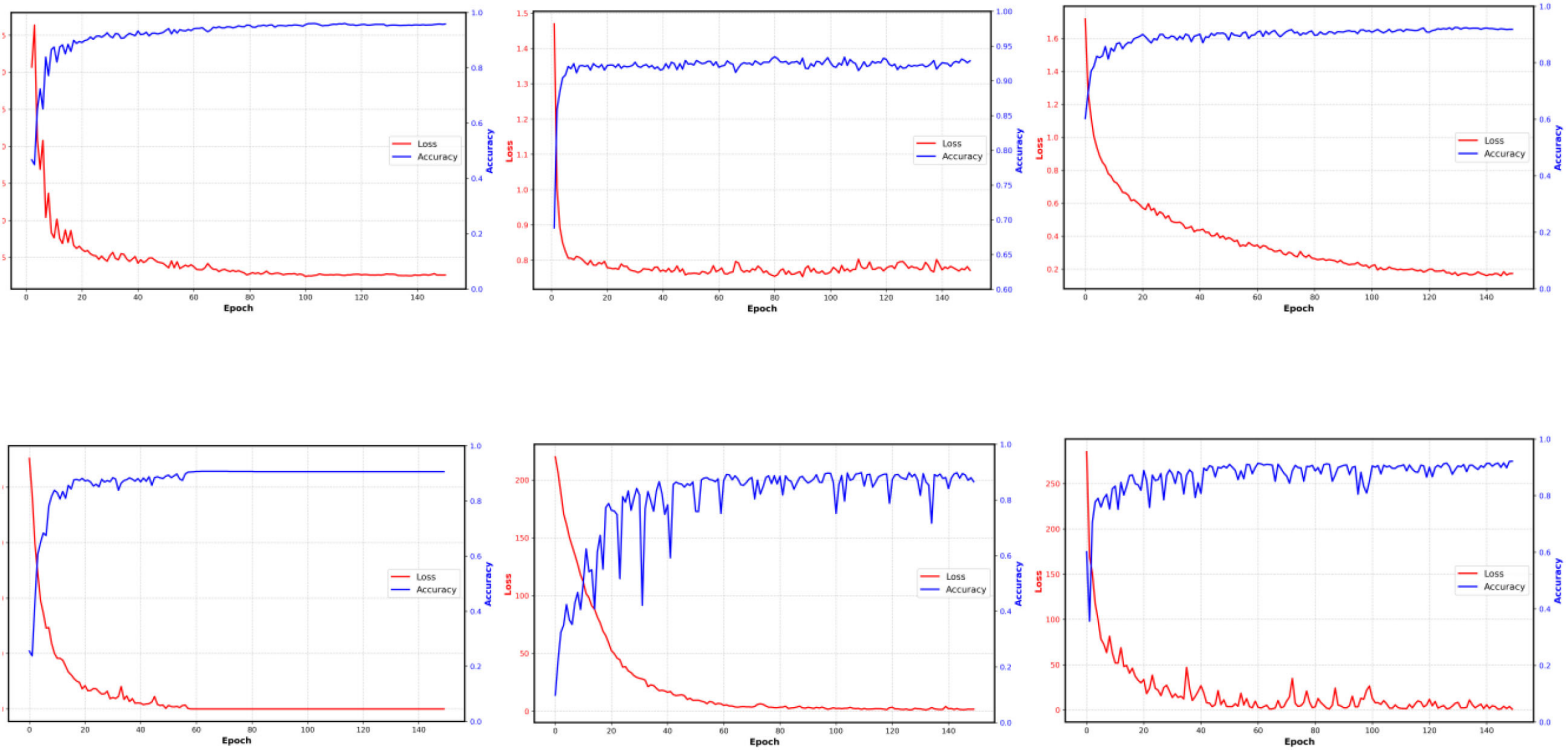
Validation accuracy and loss progress of the six models based on the PLMS feature.

**Figure 9** shows the validation accuracy and loss progress of all the used six models during the training; each line consists of 150 points, one for each epoch. As illustrated in **Figure 9**, our model rapidly reduces the loss value during the early training stages and achieves high validation accuracy within a relatively small number of iterations, maintaining stability thereafter. This demonstrates fast convergence and strong generalization capability. In comparison, although VGG19 and EfficientNetB0 demonstrate excellent convergence properties, with the loss value of VGG19 approaching zero, their final accuracy remains lower than that achieved by our model. ResNet18 also converges relatively quickly in terms of both loss and accuracy during training, yet its final accuracy still falls short. DenseNet and MobileNet, however, exhibit a slower loss decline and a more gradual accuracy increase during the initial stages of training, followed by noticeable fluctuations in the later phases. Such fluctuations may arise from their network architectures and parameter complexities, which could impede stable training. In contrast, our model excels in training efficiency, accuracy, and stability, clearly demonstrating its advantages.

### 6.6 Ablation and comparative experiments under cross-validation

As presented in Sections 6.1 to 6.5, a fixed 8:2 dataset split was employed to conduct a baseline performance comparison between our method and representative CNN architectures. While this setting offers a consistent benchmark, it may be influenced by the specific train–test partition. To further evaluate model robustness and generalization across diverse data splits, we subsequently performed a five-fold cross-validation experiment, as shown in **Table 9**. In this setting, ablation studies were performed on the proposed method, including its original version, a variant without (w/o) spectral augmentation (SpecAug), and a variant employing patchout, a regularization method that randomly discards a portion of input patches to enhance generalization. These ablation experiments were designed to isolate and quantify the contribution of each component within our framework. In parallel, we carried out comparative experiments with other algorithms, specifically EfficientNet Lite (Sangar and Rajasekar, 2025) and MobileViT (Gu et al., 2024), in order to benchmark our method against lightweight architectures proposed in recent literature. Together, these evaluations provide a more comprehensive and rigorous assessment of both accuracy and stability under varying training conditions.

**Table 9:** Ablation and comparative experiments under Cross-Validation.

Data partitioning Strategy	Algorithms	Accuracy@1	Macro-recall	Macro-F1	Macro-AUC
Fixed 8:2	**Ours**	**96.49**	**96.49**	**96.49**	**99.93**
	**Ours w/o SpecAug**	96.03	96.03	96.02	99.92
	**Ours with Patchout**	96.23	96.23	96.23	99.90
	(Sangar and Rajasekar, 2025)**:2025**	94.25	94.25	94.24	99.79
	(Gu et al., 2024)**:2024**	41.17	17.79	15.25	69.64
Five-fold cross-validation	**Ours**	**95.23±0.17**	**95.23±0.17**	**95.23± 0.17**	**99.85±0.04**
	**Ours w/o SpecAug**	95.04±0.60	95.04±0.60	95.04± 0.60	99.84±0.04
	**Ours with Patchout**	94.65±0.30	94.65±0.30	94.65± 0.30	99.85±0.02
	(Sangar and Rajasekar, 2025)**:2025**	91.46±0.75	91.41±0.75	91.43± 0.75	99.58±0.06
	(Gu et al., 2024)**:2024**	34.69±12.33	26.23± 10.89	22.4610.92	73.34±8.66

Bold values indicate the highest performance achieved by the proposed algorithm among all compared methods for each metric, including Accuracy@1, Macro-recall, Macro-F1, and Macro-AUC.

The results shown in **Figure 9** indicate that, regarding the fixed 8:2 dataset partition, our model attains the highest performance across all evaluation metrics. In contrast, the variants without SpecAug or incorporating Patchout show a marginal performance decline but still substantially outperform the baseline methods reported in references (Sangar and Rajasekar, 2025) and (Gu et al., 2024). Notably, the method in (Gu et al., 2024) exhibits significantly inferior results, with all metrics falling well below those of our model. From the perspective of five-fold cross-validation, the robustness of our model is further confirmed, consistently leading with an accuracy of 95.23% and a minimal standard deviation of 0.17, underscoring its strong generalization capability. The variant without SpecAug and the one incorporating Patchout maintain stable performance, although with slightly reduced scores. Conversely, the methods from (Sangar and Rajasekar, 2025) and (Gu et al., 2024) demonstrate comparatively poorer and more variable outcomes, particularly (Gu et al., 2024), which shows a markedly high standard deviation, indicating less stable performance. In summary, the proposed approach not only achieves superior accuracy on the fixed dataset partition, but also demonstrates exceptional stability and generalization under the more rigorous five-fold cross-validation, thereby validating the effectiveness of the model architecture and data augmentation strategies employed.

## 7 Conclusions

Early detection of pest infestations is critical for mitigating the adverse effects on agricultural productivity and ensuring ecological balance. Reviewed studies highlight the importance of balancing accuracy, scalability, and robustness in pest detection. Building upon these insights, this study presents a novel cross-modal adaptation approach for early-stage pest surveillance, utilizing the comprehensive bioacoustic InsectSound1000 database. By employing adaptive audio preprocessing, the approach effectively filters high-frequency noise and reduces computational complexity through downsampling. The utilization of PLMS spectrograms facilitates the refined transformation of acoustic signals into visual representations, enhancing the precision of time-frequency pattern extraction. The deployment of the YOLOv11 model for deep transfer learning enables the extraction of high-level features, thereby enhancing precision and the ability to generalize across diverse datasets. Experimental results demonstrate that the proposed method achieves high detection accuracy while maintaining manageable computational complexity. This framework offers a promising alternative to conventional pest monitoring techniques, paving the way for integrated, automated pest management systems that combine acoustic and visual modalities for enhanced early surveillance and pest control. However, since the InsectSound1000 dataset is collected under controlled conditions, it does not comprehensively represent practical challenges such as hardware dependency, the requirement for specialized equipment, and environmental noise encountered in real-world agricultural environments. To address these limitations, we are actively conducting field surveys and on-site experiments aimed at further validating and optimizing the proposed method for effective deployment in operational settings. Future research could focus on expanding the dataset to include a broader range of insect species and environmental conditions, which could further enhance the robustness of the model. Additionally, integrating the proposed system into existing pest management frameworks would enable automated, real-time surveillance, further optimizing pest control strategies.

## Data Availability

The original contributions presented in the study are included in the article/supplementary material. Further inquiries can be directed to the corresponding author/s.
